# Hierarchical MIL-88A/NiCo_2_O_4_@N-CNT Hybrid for Asymmetric Supercapacitors and Hydrogen Evolution Reaction

**DOI:** 10.3390/molecules31213829

**Published:** 2026-10-23

**Authors:** Jaffer Saddique, Muhammad Ahsan, Zhao Huihui, Marcin Gackowski, Łukasz Szeleszczuk, Amjad A. Almunyif

**Affiliations:** 1 Department of Physics, Qilu Institute of Technology, Jinan 250200, China; 2 Department of Toxicology and Bromatology, L. Rydygier Collegium Medicum in Bydgoszcz, Nicolaus Copernicus University in Torun, 2 Jurasza Str., 85-089 Bydgoszcz, Poland; 3 Department of Organic and Physical Chemistry, Medical University of Warsaw, 1 Banacha Str., 02-097 Warsaw, Poland; 4 Department of Physics, College of Sciences, Princess Nourah bint Abdulrahman University, P.O. Box 84428, Riyadh 11671, Saudi Arabia

**Keywords:** MIL-88A/NiCo_2_O_4_@N-CNT, asymmetric supercapacitor, specific capacity, nitrogen-doped carbon nanotubes, metal–organic frameworks, electrochemical energy storage

## Abstract

The development of multifunctional hybrid electrode materials with high-performance energy storage and electrocatalytic activity is highly desirable for advanced energy technologies. Herein, a MIL-88A/NiCo_2_O_4_@N-CNT ternary composite was prepared through ultrasonic-assisted assembly of porous MIL-88A, redox-active NiCo_2_O_4_, and conductive nitrogen-doped carbon nanotubes (N-CNTs). The synergistic interaction among these components provides abundant active sites, improved electrolyte accessibility, and enhanced charge transfer pathways. An increase in the BET surface area was observed in the case of hybrid composite (176.43 m^2^ g^−1^), compared to MIL-88A (99.35 m^2^ g^−1^) and NiCo_2_O_4_ (145.52 m^2^ g^−1^). The electrode based on MIL-88A/NiCo_2_O_4_@N-CNT was obtained with a specific capacity of 2036 C g^−1^ at 10 mV s^−1^ and 2209 C g^−1^ at 2.0 A g^−1^ in a three-electrode cell system. The specific capacity for asymmetric supercapacitors with the MIL-88A/NiCo_2_O_4_@N-CNT//AC structure was found to be 412 C g^−1^ from cyclic voltammetry (CV) and 432 C g^−1^ from galvanostatic charge–discharge (GCD), and the energy density was 98.6 Wh kg^−1^, and the power density was 1727 W kg^−1^. The electrochemical stability was also confirmed, as after 10,000 charge–discharge cycles, the hybrid supercapacitor still maintained 87.66% capacity with a coulombic efficiency of 93.60%. Moreover, the fabricated material proved to be a suitable catalyst for the hydrogen evolution reaction (HER) process, showing low overpotential (98 mV at 10 mA cm^−2^), a Tafel slope of 46.20 mV dec^−1^, and current retention of 95.3% after 20 h. This synergistic effect between the porous framework of MOF, multivalent active NiCo_2_O_4_ centers, and conducting N-CNT structure provides the above improved properties, which are effective in providing electrons.

## 1. Introduction

The emergence of portable electronics, electric cars, and other energy technologies has created the need for the development of energy storage devices, which exhibit high energy density, power density, and stability [1,2,3]. Due to their high power density, excellent cycling stability, and fast charge/discharge rates, supercapacitors can be considered as one of the potential electrochemical (EC) energy storage devices [4,5]. However, the lower energy density (ED) compared to batteries limits supercapacitor applications where a high ED is required [6]. Thus, the performance of advanced electrode materials, which offer optimized charge storage mechanisms, efficient ion/electron transport pathways, rich electroactive sites, and structural stability, is essential [7]. Recent studies on hierarchical electrode materials have further demonstrated that the integration of porous architectures with redox-active and conductive components can increase accessible active sites and facilitate ion/electron transport, thereby improving electrochemical charge storage behavior [8]. Hybrid electrode structures comprising a porous framework, a redox-active material, and a conductive carbon network have been in the limelight recently due to their potential to overcome specific drawbacks of the constituent materials, while still benefiting from their complementary advantages [9]. Metal–organic frameworks (MOFs) are crystalline structures characterized by ordered porous materials exhibiting tunable chemical compositions, large accessible surface areas, and rich coordination sites, which have been given much attention recently as promising electrode materials [10]. Such special properties facilitate the penetration of the electrolyte and conduct ion movement in EC reactions [11]. In particular, MIL-88A, which features an Fe-centered coordination environment, a flexible framework, and potential EC activity, has come to the fore as part of the various MOF families [12]. The redox processes are further possible with the presence of iron nodes, and the porous-like morphology ensures an efficient electrolyte transport and allows the utilization of the active sites [13]. Various MIL-88A-based composites have been obtained with enhanced EC aspects, modifying their structure and coating them with conductive materials [14]. As an example, MIL-88A and carbon nanotubes gave enhanced charge storage properties, including a specific capacitance (SC) of 635 F g^−1^ and ED of ~100 Wh kg^−1^, and stable cycling performance [15]. The intrinsic electrical conductivity of MIL-88A is relatively low, which slows the transfer of electrons and thus limits the extent to which electroactive sites are used [16]. Thus, it is essential to build MIL-88A-based hybrid architectures including conductive and redox-active components for improvements in EC performance to overcome such limitations [17]. Owing to their changeable oxidation states and reversible reactions, transition-metal oxides are of great interest as they display a high theoretical capacity to store charge [18]. Nickel cobalt oxide (NiCo_2_O_4_) has been given much attention due to the synergistic contribution from both Ni and Co redox couples, good electrical conductivity compared to the individual NiO and Co_3_O_4_, and a higher density of electrochemically active sites [19]. The mixed-valence state of NiCo_2_O_4_ allows for efficient charge transfer processes, rendering it a highly promising material for electrodes in a battery-type asymmetric supercapacitor [20]. Previous studies have reported the EC features of NiCo_2_O_4_, which can be greatly improved when it is incorporated into conductive carbon frameworks [21]. For example, hybrid structures of NiCo_2_O_4_ with CNTs showed better cycling stability and capacitance, thanks to better electron transport and reduced particle agglomeration [22]. In the same manner, NiCo_2_O_4_/CNT composite electrodes presented enhanced energy storage properties by offering interconnected conductive lines and promoting ion diffusion [23]. Although these benefits exist, the NiCo_2_O_4_-based electrodes still have some disadvantages such as structural instability, aggregation of active-material particles, and restriction of electroactive regions within the internal structure during deep cycles [24]. Therefore, creating hierarchical NiCo_2_O_4_-based composites with porous supports and conductive networks is still a good approach to enhance the EC performance [17].

CNTs and N-CNTs are extensively used as conducting substrates to improve the EC properties for composite electrode materials [25]. Their unique one-dimensional tubular structures, excellent electrical conductivity, high mechanical strength, and interconnected electron-transport networks enable efficient charge propagation throughout electrode architectures [26]. Moreover, the incorporation of nitrogen in the framework of CNTs introduces structural defects, which increase the surface polarity and create extra active sites, which enhance electrolyte interaction and the interfacial charge transfer process [27]. Previous studies have demonstrated that N-doped carbon nanotube-based composites can effectively enhance EC kinetics by providing continuous conductive pathways and improving the dispersion of electroactive materials [28]. For example, Wang et al. developed nitrogen-doped carbon nanotube frameworks for supercapacitor electrodes and demonstrated enhanced capacitance and rate capability due to improved electrical conductivity and increased electroactive surface sites [29]. Similarly, Yu et al. reported that nitrogen-doped carbon nanotube networks could effectively promote electron transport and stabilize transition-metal-based active materials, resulting in improved cycling performance and charge storage behavior [30]. However, because CNTs primarily contribute conductive pathways rather than intrinsic Faradaic charge storage, their integration with porous MOFs and redox-active transition-metal compounds is necessary to construct multifunctional hybrid electrodes with enhanced energy storage capability [31].

Although significant progress has been achieved in MOF-based, NiCo_2_O_4_-based, and carbon-supported hybrid electrodes, the rational integration of MIL-88A, NiCo_2_O_4_, and N-CNT into a single multifunctional architecture remains insufficiently explored. Previous studies have mainly focused on binary composites, while the synergistic contribution of a porous Fe-based MOF framework, mixed transition-metal oxide, and conductive nanotube network toward simultaneous enhancement of asymmetric supercapacitor performance and electrocatalytic activity has not been fully investigated. In this work, a hierarchical MIL-88A/NiCo_2_O_4_@N-CNT hybrid electrode was designed and synthesized by combining the complementary advantages of three functional components. The MIL-88A framework provides porous channels and structural support, NiCo_2_O_4_ contributes abundant redox-active centers, and N-CNT establishes efficient electron-transport pathways while improving structural stability. The prepared materials were systematically characterized with X-ray diffraction (XRD), Fourier-transform infrared spectroscopy (FTIR), X-ray photoelectron spectroscopy (XPS), scanning electron microscopy (SEM), and Brunauer–Emmett–Teller (BET) analysis. Furthermore, the EC performance was evaluated through cyclic voltammetry (CV), galvanostatic charge–discharge (GCD), electrochemical impedance spectroscopy (EIS), and asymmetric supercapacitor (ASC) device measurements. The hydrogen evolution reaction (HER) activity was also investigated to demonstrate the multifunctional capability of the developed hybrid electrode.

## 2. Results and Discussion

### 2.1. Structural, Textural, Spectroscopic, and Thermal Properties

**Figure 1:**
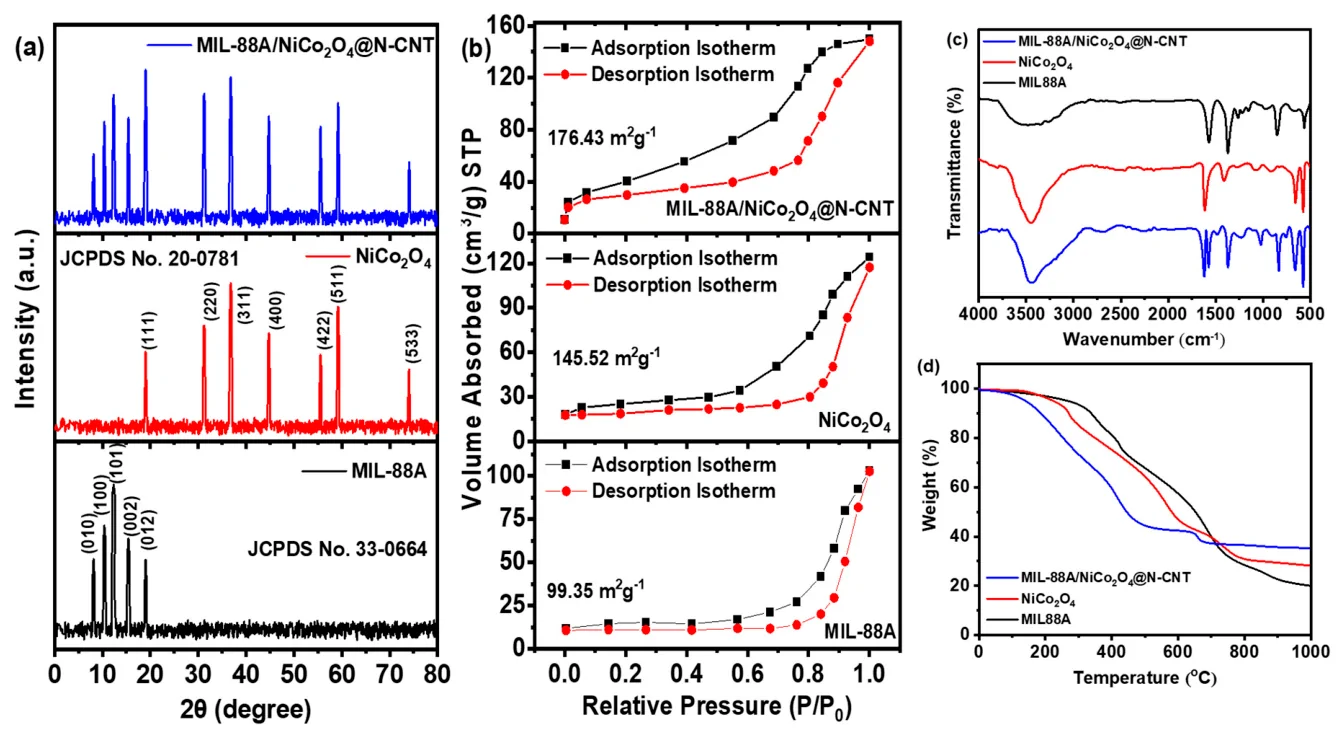
The structural, textural, and spectroscopic characteristics of the synthesized materials are shown: (**a**) XRD patterns of the synthesized materials, (**b**) N_2_ adsorption–desorption isotherms and BET specific surface areas of MIL-88A, NiCo_2_O_4_, and MIL-88A/NiCo_2_O_4_@N-CNT, (**c**) FTIR spectra of synthesized materials, and (**d**) TGA curves of MIL-88A and MIL-88A/NiCo_2_O_4_@N-CNT.

X-ray diffraction (XRD) was used to examine the crystal structure and phase composition of the prepared MIL-88A, NiCo_2_O_4_, and MIL-88A/NiCo_2_O_4_@N-CNT composite; see Figure 1a. XRD was performed to verify the successful individual component formation and to assess the structural integrity of the hybrid architecture following the composite construction. The diffraction pattern of pristine MIL-88A shows the typical reflections of the Fe-based fumarate metal–organic framework. The main diffraction peaks located at approximately 2θ = 10.0°, 10.8°, 16.4°, 18.9°, and 22.3° are indexed to the (100), (101), (110), (200), and (211) crystal planes of MIL-88A, respectively. These diffraction signals agree with the standard MIL-88A phase (JCPDS No. 33-0664), suggesting that the coordination between Fe^3+^ centers and fumarate ligands was successful and that the required porous framework [32]. Well-defined low-angle reflections are observed, suggesting that an ordered MIL-88A structure has been successfully developed. The XRD pattern of NiCo_2_O_4_ shows characteristic peaks of the cubic spinel structure. The reflections appearing at 2θ values of 18.9°, 31.2°, 36.8°, 44.8°, 59.3°, and 64.9° correspond to the (111), (220), (311), (400), (511), and (440) crystal planes, respectively. The diffraction peaks were found to be in good agreement with the JCPDS No.20-0781 of the standard spinel NiCo_2_O_4_ structure, which indicates that the NiCo_2_O_4_ was successfully synthesized as a crystalline material [33]. The lack of any impurity-induced extra peaks shows the high phase purity of the synthesized oxide material. XRD pattern of the MIL-88A/NiCo_2_O_4_@N-CNT composite shows that the diffraction peaks of the MIL-88A and NiCo_2_O_4_ phases are still present. The low-angle MIL-88A diffraction peaks coexisting with the spinel NiCo_2_O_4_ diffraction peaks further demonstrate the successful formation of the hybrid composite without the loss of the crystal structures of the respective components. In addition, there are no secondary phases identified by extra diffraction peaks, which suggests that there was no undesirable reaction during the composite fabrication process. The diffraction intensity of the composite is slightly reduced, and peak broadening occurs when compared with pristine NiCo_2_O_4_, which can be explained by the incorporation of N-CNTs and close interfacial interactions in the MIL-88A/NiCo_2_O_4_@N-CNT conductive network. Excessive crystal growth and better dispersion of the NiCo_2_O_4_ nanoparticles inside the porous MIL-88A matrix could be achieved due to the presence of N-CNTs. This structural integration is expected to help promote electrolyte ion diffusion and offer plenty of available active sites and better electron transport during EC reactions. The preservation of the characteristic diffraction features of MIL-88A and NiCo_2_O_4_, together with the absence of additional crystalline phases, indicates that ultrasonic assembly does not generate a new crystalline compound. Therefore, MIL-88A/NiCo_2_O_4_@N-CNT is described here as a physically assembled ternary composite in which the constituent phases retain their individual structural characteristics.

The porosities, specific surface areas, and adsorption properties of the synthesized MIL-88A, NiCo_2_O_4_, and MIL-88A/NiCo_2_O_4_@N-CNT composite were discussed using the N_2_ adsorption–desorption isotherm. The adsorption–desorption isotherms are shown in Figure 1b. This study was conducted to understand how the surface accessibility and the porous features change when all the components are integrated, since these will play a key role in influencing electrolyte penetration and ion transport during EC reactions. In the higher relative pressure region (P/P_0_ > 0.8), all of the prepared samples show a gradual increase in nitrogen adsorption, which shows the presence of mesopores formed by the interparticle voids and porous structure. The pristine MIL-88A sample exhibits a BET specific surface area of 99.35 m^2^ g^−1^, while NiCo_2_O_4_ shows a higher surface area of 145.52 m^2^ g^−1^. After formation of the MIL-88A/NiCo_2_O_4_@N-CNT hybrid, the BET specific surface area increases further to 176.43 m^2^ g^−1^. The BJH pore-size distribution of MIL-88A/NiCo_2_O_4_@N-CNT (Figure S1) shows a dominant pore population centered at approximately 5 nm, confirming the mesoporous nature of the composite. Furthermore, the BJH pore-size distribution presented in Figure S1 confirms the presence of accessible mesoporous/interparticle pores within the hybrid structure. The increased surface area of the MIL-88A/NiCo_2_O_4_@N-CNT hybrid is attributed to the hierarchical organization produced during assembly of the three components. The N-CNT network provides a one-dimensional supporting scaffold that improves the dispersion of NiCo_2_O_4_ nanoparticles and restricts excessive particle aggregation. In addition, the interfaces among MIL-88A, NiCo_2_O_4_, and N-CNTs generate additional interparticle voids and interconnected porous pathways, thereby exposing surfaces that may otherwise remain inaccessible in the individual components. Therefore, the N-CNT contribution is primarily associated with improved particle dispersion, formation of interfacial porosity, and increased accessibility of the hybrid surface rather than a simple additive increase in surface area. The resulting hierarchical porous network is favorable for electrolyte penetration, ion diffusion, and accessibility of electrochemically active sites. Moreover, the interconnected porous structure provides shorter ion/electron transport pathways, which is expected to contribute to improved charge storage behavior and rate capability of the hybrid electrode.

The chemical bonding nature and surface functional groups of MIL-88A, NiCo_2_O_4_, and MIL-88A/NiCo_2_O_4_@N-CNT were explored by FTIR analysis. The results are shown in Figure 1c. The FTIR spectrum of MIL-88A exhibits a broad O–H stretching band around 3400 cm^−1^, together with the asymmetric and symmetric stretching vibrations of the fumarate carboxylate groups at approximately 1550–1600 and 1390–1450 cm^−1^, respectively. The C–O stretching vibration of the organic ligand is observed in the region of 1000–1100 cm^−1^. For NiCo_2_O_4_, the characteristic metal–oxygen vibrations occur at approximately 650–700 cm^−1^ and 500–550 cm^−1^, corresponding to Co–O and Ni/Co–O vibrations of the spinel structure. The MIL-88A/NiCo_2_O_4_@N-CNT composite retains these characteristic bands, with minor variations in peak position and intensity compared with the individual components. These changes are consistent with interfacial association among the assembled components, possibly involving oxygen-containing functional groups at the MIL-88A/NiCo_2_O_4_/N-CNT interfaces. The interactions are useful for enhancing the electron transfer pathways and structural stability in EC cycling.

The thermal stability and decomposition of MIL-88A without modification and MIL-88A/NiCo_2_O_4_@N-CNT were studied using TGA, and the results are shown in Figure 1d. The TGA profiles are important for data concerning the removal of physically adsorbed species, decomposition of organic components, and thermal stability of the resulting hybrid structure. The TGA curve of MIL-88A shows a multi-step weight loss. This initial weight loss below 150 °C is primarily due to the loss of the physically adsorbed water molecules, and also to the loss of the solvent molecules physically trapped inside the porous framework. This is followed by a significant weight loss (250–450 °C) that is most probably ascribed to the volatilization of the fumarate organic linkers and the collapse of the framework of MIL-88A. At temperatures above this range, the remaining mass is due to the formation of thermally stable residues of iron oxide, which form after complete decomposition of the organic moieties. The composite of MIL-88A/NiCo_2_O_4_@N-CNT, on the other hand, exhibits a higher thermal stability and a lower degradation rate, even as high as 80% residual mass at the end of the heating process. The temperature dependence of initial weight loss is correlated with the removal of surface-bound species and/or adsorbed moisture. The partial weight loss around the intermediate temperature is related to the decomposition of the MIL-88A organic framework and the release of oxygen-containing surface functional groups from N-CNTs. This is due to the relatively higher residual mass at elevated temperatures resulting from the presence of the inorganic phase NiCo_2_O_4_, which is thermally stable, and the carbonaceous N-CNT network that survives the decomposition of the organic components. The hybrid composite exhibits delayed thermal degradation, as compared to pristine MIL-88A, demonstrating the enhanced thermal stability of the composite because of the synergistic interaction between the MOF framework, NiCo_2_O_4_ nanoparticles, and the conductive N-CNT network. The addition of NiCo_2_O_4_ and N-CNTs not only improves the thermal resistance of the composite, but also gives the composite a stable interconnected structure, which is beneficial to the stability of the electrode structure in repeated EC charge/discharge processes. The successful preparation of the MIL-88A/NiCo_2_O_4_@N-CNT hybrid structure is therefore confirmed by the TGA results, where it is found that the presence of inorganic NiCo_2_O_4_ and carbon nanotubes greatly enhances the thermal stability of the composite material in comparison to the pure MIL-88A framework.

### 2.2. X-Ray Photoelectron Spectroscopy (XPS) Analysis

**Figure 2:**
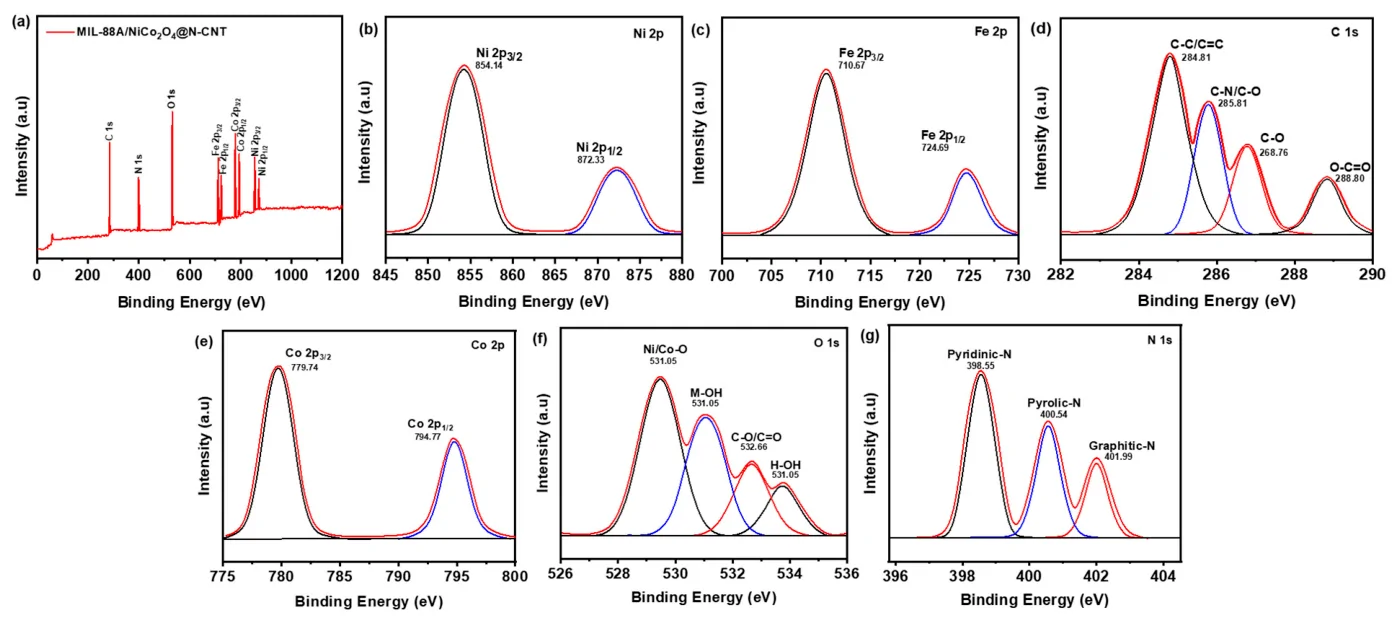
XPS analysis of the MIL-88A/NiCo_2_O_4_@N-CNT composite: (**a**) XPS survey spectrum, (**b**) high-resolution Ni 2p spectrum, (**c**) high-resolution Fe 2p spectrum, (**d**) deconvoluted C 1s spectrum, (**e**) high-resolution Co 2p spectrum, (**f**) deconvoluted O 1s spectrum, and (**g**) N 1s spectrum.

The surface elements, their oxidation states, and bonding nature of the MIL-88A/NiCo_2_O_4_@N-CNT composite material were analyzed using X-ray photoelectron spectroscopy (XPS), and the corresponding spectra are shown in Figure 2. Based on the survey spectrum (Figure 2a), one can observe that Fe, Ni, Co, C, and O are the components of the composite material, which reveals the incorporation of MIL-88A, NiCo_2_O_4_, and N-CNT into the structure of the composite material. No other peaks of impurities have been seen. The high-resolution Ni 2p spectrum (Figure 2b) exhibits the characteristic spin–orbit splitting peaks associated with Ni species. The Ni 2p_3_/_2_ and Ni 2p_1_/_2_ peaks are located at approximately 854.5 eV and 872.2 eV, respectively, accompanied by corresponding satellite peaks at higher binding energies. These features confirm the coexistence of Ni^2+^/Ni^3+^ oxidation states in the spinel NiCo_2_O_4_ structure. The presence of multiple valence states is beneficial for EC reactions because the reversible conversion between Ni oxidation states provides additional faradaic active sites during charge storage. The Fe 2p spectrum (Figure 2c) displays two major characteristic peaks corresponding to Fe 2p_3_/_2_ and Fe 2p_1_/_2_ located around 711.0 eV and 724.5 eV, respectively. The separation between these peaks, together with the associated satellite features, confirms the presence of Fe species derived from the MIL-88A framework. The Fe-based metal centers are responsible for providing additional redox-active sites and contribute to the pseudocapacitive behavior of the hybrid electrode. One of the C 1s spectra deconvoluted (see Figure 2d) shows several carbon-containing peaks, indicating that the compound has different binding sites for carbon. The dominant peak at approximately 284.8 eV is assigned to graphitic C=C/C–C bonding originating from the N-CNT conductive network. Additional peaks located near 286.2 eV and 288.5 eV correspond to oxygen-containing functional groups such as C–O and O–C=O, respectively. They enhance the wettability of the electrolyte and the interaction between the ion and the electrode surface. The high-resolution Co 2p spectrum (Figure 2e) shows two dominant peaks at 779.8 eV (Co 2p_3_/_2_) and 795.0 eV (Co 2p_1_/_2_) accompanied by some satellite peaks. From the observed peak positions, it is assumed that the NiCo_2_O_4_ phase has mixed oxidation states of Co^2+^/Co^3+^.The coexistence of multiple cobalt oxidation states enhances the EC activity by enabling rapid reversible redox transitions during charge/discharge processes. The fitted O 1s spectrum (Figure 2f) consists of three oxygen contributions. The peak located around 529.5 eV corresponds to metal–oxygen bonds (M–O, M = Fe, Ni, and Co) associated with the metal oxide framework. The peak near 531.2 eV is assigned to surface hydroxyl groups and defect-related oxygen species, while the higher binding energy component around 532.5 eV originates from adsorbed oxygen-containing molecules or surface functional groups. The presence of oxygen vacancies and surface-active oxygen species can provide additional reaction sites and promote electrolyte ion adsorption. The high-resolution N 1s spectrum (Figure 2g) provides additional evidence for the nitrogen-containing carbon component in the MIL-88A/NiCo_2_O_4_@N-CNT composite. The N 1s spectrum can be deconvoluted into contributions centered at approximately 398.55, 400.54, and 401.99 eV, assigned to pyridinic-N, pyrrolic-N, and graphitic-N species, respectively. Together with the graphitic carbon contribution observed in the C 1s spectrum, these nitrogen species support the incorporation of the N-CNT component into the assembled composite. The presence of the N 1s signal, together with the graphitic carbon contribution in the C 1s spectrum, provides complementary chemical evidence for the incorporation of N-CNTs in the MIL-88A/NiCo_2_O_4_@N-CNT composite. The coexistence of Fe, Ni, and Co multivalent species together with oxygen defect sites is expected to accelerate electron transfer kinetics and enhance faradaic charge storage behavior, contributing to the improved EC performance of the composite electrode.

### 2.3. Morphological Analysis

**Figure 3:**
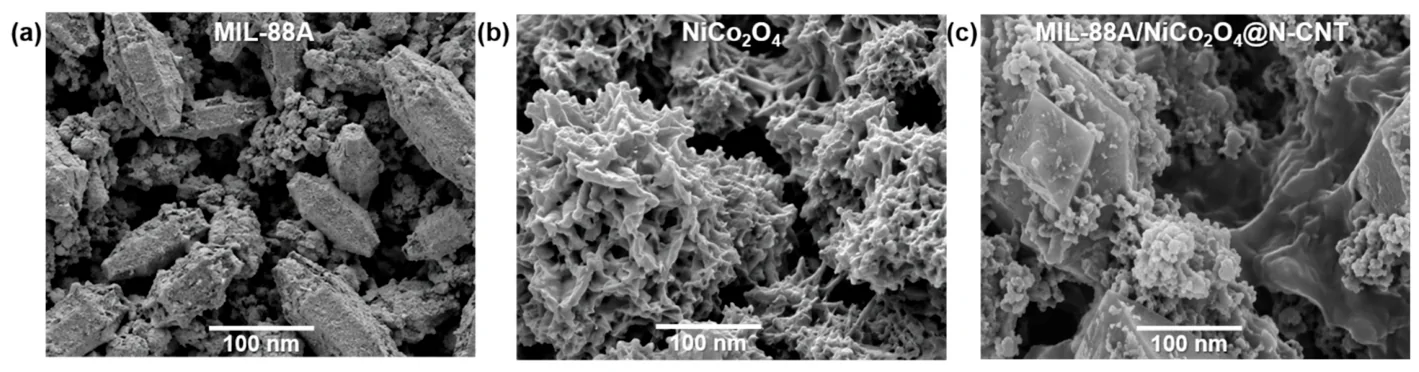
SEM images of (**a**) MIL-88A, (**b**) NiCo_2_O_4_, and (**c**) MIL-88A/NiCo_2_O_4_@N-CNT composite at a scale of 100 nm.

The surface morphology and structural features of the synthesized materials were investigated by scanning electron microscopy (SEM), and the corresponding images of pristine MIL-88A, NiCo_2_O_4_, and MIL-88A/NiCo_2_O_4_@N-CNT composite are presented in Figure 3. Direct evidence of successful integration of the individual components and morphological evolution after composite formation is evident in the SEM images. The pure MIL-88A sample has a well-defined elongated rod-like shape, smoothness, and uniformity as displayed in Figure 3a. The unique 1D topology of MIL-88A is attributed to the anisotropic growth pattern of the metal–organic framework throughout the solvothermal synthesis. This porous, rod-like structure can allow the electrolyte to penetrate the channel and allow the diffusion of ions during EC reactions. The shape and morphology of the NiCo_2_O_4_ nanoparticles are clearly visible from the SEM image (Figure 3b), where interconnected spherical particles at the nano level are observed. The small size of NiCo_2_O_4_ nanoparticles and the high surface accessibility result in a large number of exposed active sites. The tendency of aggregation of single NiCo_2_O_4_ nanoparticles, however, partly hinders electron transport and ion diffusion, making the need for forming a hybrid structure of conductive carbon support essential. The SEM image of the hybrid composite of MIL-88A/NiCo_2_O_4_@N-CNT (Figure 3c) vividly illustrates a well-defined integrated structure featuring NiCo_2_O_4_ nanoparticles uniformly embedded within the MIL-88A framework and interconnected with the N-CNT network. The presence of MIL-88A nanorods and the uniform distribution of NiCo_2_O_4_ nanoparticles show successful coupling between the MOF and the metal oxide components. In addition, the N-CNTs exist as a continuous conductive network around the active materials, thus suppressing nanoparticle aggregation and providing continuous electron conduction pathways. However, owing to the resolution limitation of SEM and the overlapping carbon-containing components, the N-CNTs and their nanoscale interfacial contacts with MIL-88A and NiCo_2_O_4_ cannot be unambiguously resolved from the SEM image alone. Therefore, Figure 3c is interpreted only as evidence of morphological integration rather than direct confirmation of the proposed nanoscale architecture. The presence of the N-CNT component is additionally supported by the C 1s and newly added N 1s XPS spectra. The hierarchical composite structure generated by combining porous MIL-88A, redox-active NiCo_2_O_4_ nanoparticles, and conductive N-CNTs is expected to improve electrolyte accessibility, shorten ion diffusion pathways, and enhance charge transfer kinetics.

## 3. Experimental Section

### 3.1. Materials and Chemicals

Iron(III) chloride hexahydrate (FeCl_3_·6H_2_O, ≥99%), fumaric acid (C_4_H_4_O_4_, ≥99%), nickel nitrate hexahydrate (Ni(NO_3_)_2_·6H_2_O, ≥98%), cobalt nitrate hexahydrate (Co(NO_3_)_2_·6H_2_O, ≥98%), nitrogen-doped carbon nanotubes (N-CNTs, ≥95%), activated carbon (AC, ≥99%), polyvinylidene fluoride (PVDF, ≥99%), and N-methyl-2-pyrrolidone (NMP, ≥99.5%) were obtained from Sigma-Aldrich (Louis, LA, USA) and used as received without further purification. Potassium hydroxide (KOH, ≥85%) was used as an electrolytic medium for EC measurements. Hydrochloric acid (HCl, ≥37%) and ethanol (≥99.5%) were used during the synthesis and washing processes. Deionized water (DIW) was used throughout all experimental procedures.

### 3.2. Synthesis of MIL-88A

MIL-88A was synthesized through a solvothermal method. Typically, FeCl_3_·6H_2_O (1 mmol, 270.3 mg) and fumaric acid (1 mmol, 116.1 mg) were dissolved in a mixed solvent containing 20 mL N,N-dimethylformamide (DMF) and 5 mL DIW under continuous stirring for 30 min to obtain a homogeneous precursor solution. The resulting solution was transferred into a 50 mL Teflon-lined stainless-steel autoclave (TLSSA) and heated at 120 °C for 12 h, which was followed by the cooling process at 300 K (room temperature). The unreacted chemicals were separated by centrifugation and subsequently washed with DMF and ethanol to obtain the yellow product. The purified sample was then allowed to dry for 12 h under vacuum at 60 °C to obtain the MIL-88A powder.

### 3.3. Synthesis of NiCo_2_O_4_

NiCo_2_O_4_ was synthesized through a hydrothermal-assisted calcination process. In general, nickel nitrate hexahydrate Ni(NO_3_)_2_·6H_2_O (1 mmol, 290.8 mg) and cobalt nitrate hexahydrate Co(NO_3_)_2_·6H_2_O (2 mmol, 582.0 mg) were allowed to mix in 40 mL of DIW under constant stirring. Subsequently, urea (6 mmol, 360.4 mg) was introduced as a precipitating agent, and the resulting mixture was allowed to stir up to 1 h to obtain uniformity. The prepared solution was subsequently transferred into a 50 mL autoclave (Teflon) and allowed to stand for 12 h at 120 °C. Then, washing using DIW/ethanol and drying for 10–12 h at a temperature of 80 °C were followed by solidification of the precursor. The dried sample was then heated again in an air atmosphere at 350 °C for 2 h at 2 °C/min to form the crystalline NiCo_2_O_4_.

### 3.4. Synthesis of MIL-88A/NiCo_2_O_4_@N-CNT Composite

**Figure 4:**
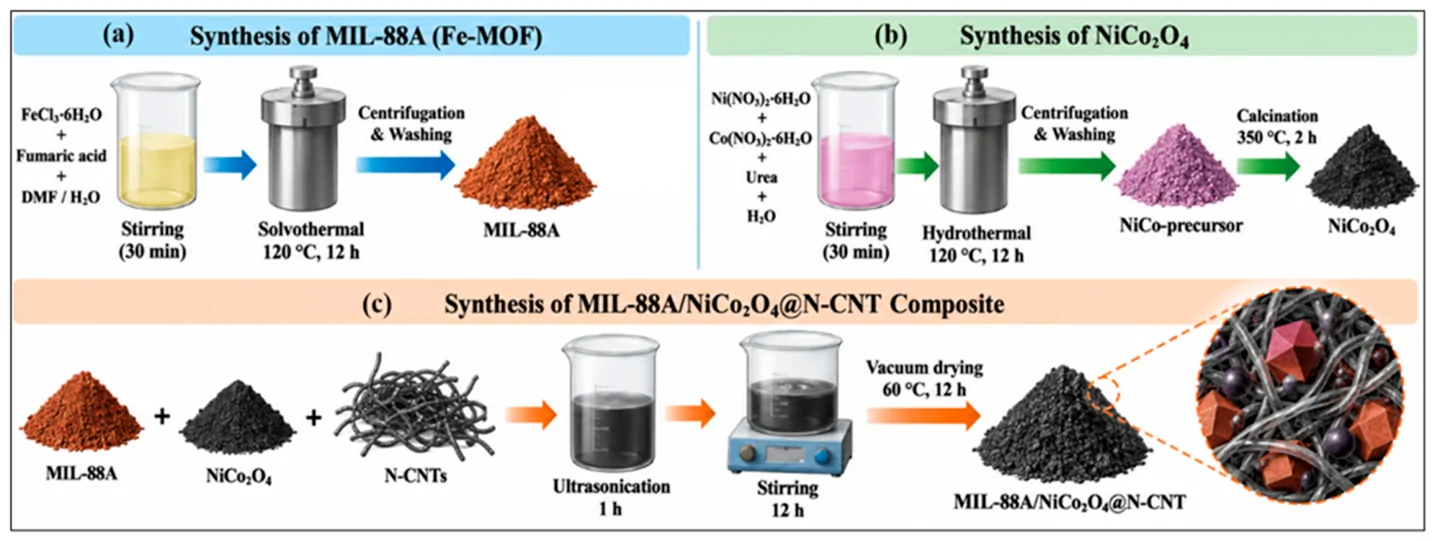
Schematic illustration of the synthesis routes of (**a**) MIL-88A, (**b**) NiCo_2_O_4_, and (**c**) MIL-88A/NiCo_2_O_4_@N-CNT composite.

The MIL-88A/NiCo_2_O_4_@N-CNT ternary composite was prepared through an ultrasonic-assisted physical assembly strategy. Briefly, MIL-88A (0.5 g), NiCo_2_O_4_ (0.5 g), and nitrogen-doped carbon nanotubes (0.1 g) were finely distributed in 50 mL ethanol under ultrasonication for 1 h to facilitate the homogeneous dispersion of the individual constituents. The obtained suspension was continuously stirred for 12 h to promote intimate interaction among the porous MIL-88A framework, NiCo_2_O_4_ nanoparticles, and conductive N-CNT network. After completion, the composite was separated by centrifugation, repeatedly washed with ethanol, and then allowed to dry at 60 °C for 12 h. The obtained ternary composite was denoted as MIL-88A/NiCo_2_O_4_@N-CNT. The rationally designed composite architecture combines the advantages of each component: MIL-88A provides a porous architecture, which supports the electrolytic accessibility and ion diffusion, NiCo_2_O_4_ contributes abundant reversible redox-active sites, and N-CNT establishes conductive pathways to facilitate electron transport and improve structural stability. For comparison, pristine MIL-88A and NiCo_2_O_4_ samples were prepared using the same procedures and evaluated under identical EC conditions (Figure 4).

### 3.5. Material Characterization

Various characterization techniques have been used to study the geometrical and morphological traits as well as surface chemistry aspects of the prepared nanocomposites. The crystal structures of MIL-88A, NiCo_2_O_4_, and MIL-88A/NiCo_2_O_4_@N-CNT hybrid material were determined by XRD using the Cu Kα irradiation having wavelength λ = 1.5406 Å at the angular range of 10–80°. FTIR was used to signify the functional groups in the material across the 400 to 4000 cm^−1^ wavenumber range. SEM analysis was used to gain deeper insight into the surface morphology and microstructure of the materials. XPS technique was used to evaluate the chemical states and oxidation states of the individual elements. The obtained XPS spectra were calibrated using the C 1s peak at 284.8 eV. Specific surface area (SA) and pore volume were estimated by studying the nitrogen adsorption–desorption isotherm using the BET method. The obtained structural information was used to understand the influence of the hybrid architecture on surface accessibility and electrolyte transport behavior.

### 3.6. Electrode Fabrication and Electrochemical Measurements

The EC electrodes were fabricated using a conventional slurry-coating method. The synthesized MIL-88A/NiCo_2_O_4_@N-CNT composite, pristine MIL-88A, and NiCo_2_O_4_ powders were used as active electrode materials. In general, the ratio of the active materials (AM) to carbon black to PVDF binder was 80:10:10 (weight ratio) to ensure that there was good electrical conductivity and adhesion. The active-material loadings on the 1 × 1 cm^2^ nickel foam substrates were 1.80, 1.95, and 2.10 mg cm^−2^ for MIL-88A, NiCo_2_O_4_, and MIL-88A/NiCo_2_O_4_@N-CNT, respectively. Later, N-Methyl-2-Pyrrolidone (NMP) was used as the solvent, and then the solution was stirred overnight.

The uniform slurry was evenly coated on the surface of pre-cleaned nickel foam (NF, 1 × 1 cm^2^) and then dried at 60 °C under a vacuum condition for 12 h to remove the solvent. The dried electrodes were further pressed to improve the contact between the AM and nickel foam current collector. The amount of AM loaded was calculated according to Equation (1):

(1) $$M_{L}=\frac{m_{a}}{A}$$

where $M_{L}$ represents the AM mass (mg cm^−2^), $m_{a}$ is the AM mass, which is coated on the nickel foam (mg), and A is the geometric area of the electrode (cm^2^).

The fabricated MIL-88A/NiCo_2_O_4_@N-CNT/NF electrode was directly employed as the positive electrode for EC measurements. For comparison, MIL-88A/NF and NiCo_2_O_4_/NF electrodes were fabricated using the same synthesis methodology under identical conditions. For the asymmetric supercapacitor (ASC) device, the MIL-88A/NiCo_2_O_4_@N-CNT composite electrode was selected as the +ve electrode, while the activated carbon (AC)-based electrode served as the −ve electrode. The balance of mass between the +ve and −ve electrodes was optimized using the charge balance principle:

(2) $$q^{+}=q^{-}$$

(3) $$C^{+}m^{+}\Delta V^{+}=C^{-}m^{-}\Delta V^{-}$$

$C^{+}$/$C^{-}$ are the specific charge capacities (SCC) of the +ve/−ve electrodes (C g^−1^), $m^{+}$ and $m^{-}$ represent the masses of active materials on the +ve and −ve electrodes (g), respectively, and $\Delta V^{+}$ and $\Delta V^{-}$ correspond to the potential windows of the individual electrodes. Following the optimized mass balance, the ASC device was assembled using MIL-88A/NiCo_2_O_4_@N-CNT as the cathode and activated carbon as the anode with a suitable separator and alkaline electrolyte.

### 3.7. Electrochemical Measurements

In a three-electrode system, the EC properties of the MIL-88A, NiCo_2_O_4_, and MIL-88A/NiCo_2_O_4_@N-CNTs electrodes were measured in a 6 M aqueous KOH electrolyte solution at 300 K. The prepared electrode materials, which were coated on nickel foam, were used as working electrodes, while platinum foil and Hg/HgO electrode were used as counter and reference electrodes, respectively. All EC experiments were carried out with a potentiostat/galvanostat EC workstation.

The CV curves were acquired at various scan rates in order to investigate the reversibility of the redox reactions. The specific charge capacity ($Q_{s}$) obtained from CV curves was calculated according to the following equation:

(4) $$Q_{s,CV}=\frac{\int_{V_{1}}^{V_{2}}I\left(V\right)dV}{m\nu }$$

Here, $Q_{s,CV}$ is attributed to the SCC (C g^−1^), $I\left(V\right)$ is the instantaneous current response, m is the mass of AM, and ν represents the scan rate.

To evaluate the charge storage capability, galvanostatic charge–discharge (GCD) measurements at different current densities were carried out. The SCC derived from GCD curves was calculated using the following equation:

(5) $$Q_{s,GCD}=\frac{I\Delta t}{m}$$

where I and Δt represent the discharge current (A) and time (s), and m represents the mass loading of AM (g).

For the asymmetric supercapacitor device, the charge balance between the +ve and −ve electrodes was maintained according to the following equation:

(6) $$m_{+}Q_{+}=m_{-}Q_{-}$$

where $m_{+}$/$m_{-}$ represent the mass loading of +ve/−ve electrodes, while $Q_{+}$ and $Q_{-}$ correspond to their respective specific charge capacities.

The required mass ratio between +ve and −ve electrodes was calculated using the following equation:

(7) $$\frac{m_{+}}{m_{-}}=\frac{Q_{-}}{Q_{+}}$$

The EC effectiveness of the assembled asymmetric device was further evaluated by calculating the ED and PD. The ED was determined using the following equation:

(8) $$E=\frac{Q_{s}\Delta V}{2\times 3.6}$$

where E represents the ED (Wh kg^−1^), $Q_{s}$ is the SCC (C g^−1^), and ΔV represents the operating voltage window.

The corresponding PD was calculated according to the following equation:

(9) $$P=\frac{3600E}{\Delta t}$$

where P indicates the PD (W kg^−1^), and Δt is the discharge time (s).

The specific charge capacity of the asymmetric device was obtained directly from the experimental galvanostatic charge–discharge profile using Q_s_ = IΔt/m, where Q_s_ is the specific charge capacity (C g^−1^), I is the discharge current (A), Δt is the discharge time (s), and m is the active-material mass (g). Because the positive electrode exhibits battery-type/Faradaic charge storage behavior and consequently a non-linear discharge response, the device energy density was estimated from the experimentally measured specific charge capacity and the established operating voltage window rather than by deriving an ideal specific capacitance from the discharge slope. The energy density was calculated using E = Q_s_ΔV/3600, where E is the energy density (Wh kg^−1^), Q_s_ is the experimentally determined specific charge capacity (C g^−1^), and ΔV is the operating voltage window (V). The corresponding power density was calculated according to P = 3600E/Δt, where P is the power density (W kg^−1^), and Δt is the experimental discharge time (s).

The charge storage kinetics were further analyzed by studying the response of current with the following scan rate:

(10) $$i=av^{b}$$

(11) $$log\left(i\right)=blog\left(v\right)+log\left(a\right)$$

where i represents the peak current, v is the scan rate, a is a constant, and b is the kinetic parameter. The b-value was obtained from the slope of the linear relationship between $log\left(i\right)$ and $log\left(v\right)$. A b-value close to 1 indicates a surface-controlled capacitive process, whereas a value approaching 0.5 suggests diffusion-controlled ion storage.

To further separate the capacitive and diffusion-controlled contributions, the current response was separated according to the following equation:

(12) $$i\left(V\right)=k_{1}v+k_{2}v^{1/2}$$

$k_{1}$v corresponds to the capacitive-controlled contribution, and $k_{2}v^{1/2}$ represents the diffusion-controlled contribution.

EC impedance spectroscopy (EIS) was performed over a frequency range of 100 kHz–0.01 Hz with a small-amplitude AC perturbation to analyze the electrode/electrolyte interface characteristics. The Nyquist plots were used to determine the equivalent series resistance ($R_{s}$), charge transfer resistance ($R_{ct}$), and ion diffusion behavior.

Long-term cycling stability was evaluated through repeated GCD charge–discharge cycles. The capacity retention was calculated by comparing the capacity before and after cycling:

(13) $$\mathrm{Retention}\;\left(\%\right)=\frac{C_{n}}{C_{0}}\times 100$$

where $C_{0}$ and $C_{n}$ are the initial and final capacity values, respectively.

### 3.8. HER Electrocatalytic Measurements

The electrocatalytic activity of the MIL-88A, NiCo_2_O_4_, and MIL-88A/NiCo_2_O_4_@N-CNT electrodes was examined by a standard three-electrode system EC setup in the reaction of hydrogen evolution reaction (HER). The prepared catalyst-coated electrode was used as the working electrode, while a platinum wire and Hg/HgO electrode were employed as the counter and reference electrodes, respectively. All HER measurements were conducted in 1.0 M KOH aqueous electrolyte using an EC workstation.

For the preparation of HER working electrodes, a catalyst ink was prepared by uniformly dispersing the synthesized material in a mixture of ethanol and Nafion solution under ultrasonication. The obtained homogeneous suspension was drop-cast onto a conductive substrate (Ni foam) and dried at room temperature before EC testing. The catalyst loading was maintained constant for all electrodes to ensure a reliable comparison of electrocatalytic activity. To assess the HER catalytic activity, linear sweep voltammetry (LSV) measurements were carried out at a scan rate of 5 mV s^−1^. All polarization curves were compensated for ohmic resistance (iR compensation), and the potentials measured were converted to the reversible hydrogen electrode (RHE) scale:

(14) $$E_{RHE}=E_{Hg/HgO}+0.0591\times pH+E_{Hg/HgO}^{0}$$

where $E_{RHE}$ is the potential relative to the reversible hydrogen electrode, $E_{Hg/HgO\;}$ is the experimentally measured potential against the Hg/HgO reference electrode, and $E_{Hg/HgO\;}^{0}$ represents the standard potential of the reference electrode.

The evaluation of catalytic activity of HER was performed by the overpotential needed to attain a current density of 10 mA cm^−2^ (η_10_). A smaller overpotential means greater hydrogen evolution kinetics and catalytic efficiency. The linear range of the Tafel plot was used to determine the Tafel slope as follows:

(15) $$\eta =blog\left(j\right)+a$$

The overpotential, η (mV), is the current density, j (mA cm^−2^), multiplied by the Tafel slope, b, plus the intercept, a. The Tafel slope obtained was analyzed to understand the HER reaction kinetics, which is smaller the more favorable the reaction kinetics. The stability of HER was studied over a longer period of time, using chronoamperometric measurements at a constant applied potential. The retention of catalytic current over extended operation was used to evaluate the durability of the catalyst. The morphology and structure of the electrodes were then examined after stability testing, in order to verify the stability of the MIL-88A/NiCo_2_O_4_@N-CNT hybrid architecture.

The synergistic effect of the three components: the porous framework of MIL-88A allows the penetration of the electrolyte; NiCo_2_O_4_ offers a large number of transition-metal active sites for the water reduction reaction process of the HER; and N-CNT has better electrical conductivity and high-speed electron transfer rate during the HER process.

## 4. Electrochemical Performance

### 4.1. Three-Electrode Measurements

**Figure 5:**
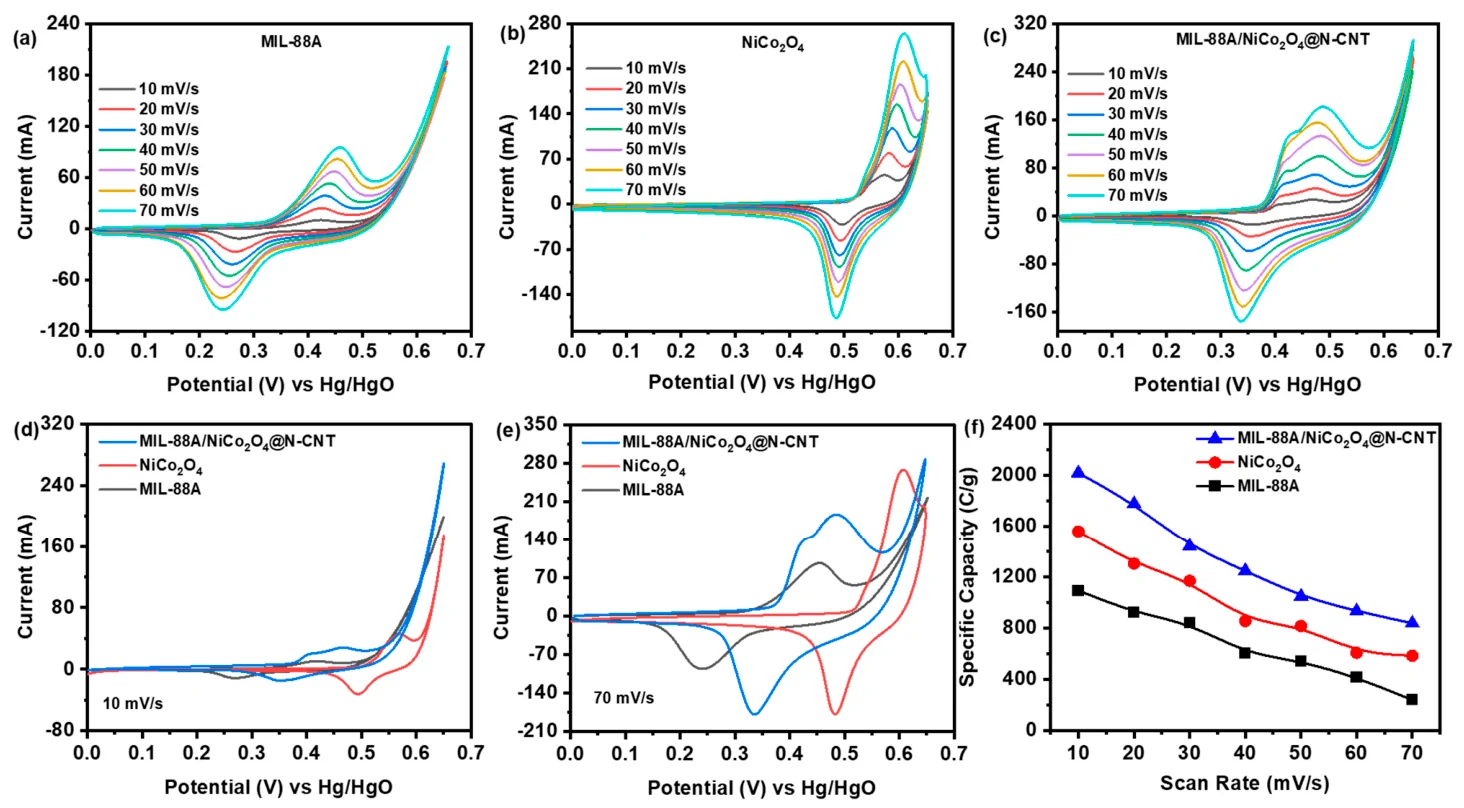
Electrochemical activity of MIL-88A, NiCo_2_O_4_, and MIL-88A/NiCo_2_O_4_@N-CNT electrodes: CV curves of (**a**) MIL-88A, (**b**) NiCo_2_O_4_, and (**c**) MIL-88A/NiCo_2_O_4_@N-CNT at different scan rates from 10 to 70 mV s^−1^; comparative CV curves of the three electrodes at (**d**) 10 mV s^−1^ and (**e**) 70 mV s^−1^; and (**f**) specific capacity as a function of scan rate for MIL-88A, NiCo_2_O_4_, and MIL-88A/NiCo_2_O_4_@N-CNT.

Charge storage capacity measurements were carefully examined by the CV method in three-electrode system setups for the fabricated MIL-88A, NiCo_2_O_4_, and MIL-88A/NiCo_2_O_4_@N-CNT electrodes. Figure 5a–c displays the CV profiles of all three electrodes at different scan rates ranging from 10 to 70 mV s^−1^, which are significantly different from the rectangular CV curves of an ideal EDC. Faradaic reactions are shown in the charge storage process through the presence of the anodic and cathodic redox peaks. The CV curves of MIL-88A (Figure 5a) exhibit characteristic redox responses originating from the reversible oxidation/reduction reactions of Fe-based active centers within the porous metal–organic framework. With increasing scan rate, the anodic and cathodic peak currents gradually increase, demonstrating enhanced EC response under higher charge–discharge conditions. The EC utilization of MIL-88A is, however, limited by its low electrical conductivity, as suggested by the relatively moderate current response. In the case of the NiCo_2_O_4_ electrode (Figure 5b), more prominent redox peaks are observed than those of MIL-88A, which can be attributed to the multiple oxidation states and a large number of electroactive sites of Ni and Co ions. The reversible Ni^2+^/Ni^3+^ and Co^2+^/Co^3+^ transitions contribute significantly to the faradaic charge storage process. With the increase in scan rate, the increasing peak current signifies the good EC activity of NiCo_2_O_4_, but some overlap and polarization occur at higher scan rates, suggesting that there are still ion diffusion and electrical transport limitations. On the other hand, when compared to other electrodes, the electrochemical activity is found to be quite high for the hybrid electrode MIL-88A/NiCo_2_O_4_ (Figure 5c), which has the highest redox peak intensity (Figure 5a,b). The anodic and cathodic peaks in the well-preserved sample are excellent examples of the reversibility and better reaction kinetics at a high scan rate of 70 mV s^−1^. This excellent performance is due to the synergistic combination of the three components. The electron-porous MIL-88A structure allows for easy access to the electrolyte, the nanoparticles of NiCo_2_O4 offer plenty of reversible redox sites, and the N-CNT network creates conductive paths for the rapid transport of electrons. The synergistic structure benefits lead utilization and speeds up the faradaic reaction.

The CV curves of all the electrodes at 10 mV s^−1^ and 70 mV s^−1^ are shown in Figure 5d and Figure 5e, respectively, for comparison of the electrochemical behavior. The MIL-88A/NiCo_2_O_4_@N-CNT electrode has a significantly greater integrated CV area than both the pristine MIL-88A and NiCo_2_O_4_ electrodes at both scan rates, demonstrating enhancement of the charge storage capacity. This is because the hybrid architecture increases the number of electroactive sites that are accessible and increases the charge transfer efficiency. The specific capacities of the MIL-88A, NiCo_2_O_4_, and MIL-88A/NiCo_2_O_4_@N-CNT electrodes at the lowest scan rate (10 mV s^−1^) are shown to be 1113, 1596, and 2036 C g^−1^, respectively (as summarized in Figure 5f). The effectiveness of the hybrid design is verified by the superior capacity of the MIL-88A/NiCo_2_O_4_@N-CNT electrode, which is superior to that of the individual components. The enhanced charge storage performance arises from the complementary contributions of the Fe-based redox-active sites of MIL-88A, the reversible Ni^2+^/Ni^3+^ and Co^2+^/Co^3+^ redox couples of NiCo_2_O_4_, and the highly conductive N-CNT network. The carbon nanotube network brings about improved electrical conductivity and decreased charge transfer resistance, and the porous hybrid structure allows the penetration of the electrolyte and facilitates quick ion transport. Thus, the reversible electrochemical reactions involve a larger number of active sites, which leads to the highest specific capacity of 2036 C g^−1^. The overall CV results confirm that the rational combination of the three components, namely, MIL-88A, NiCo_2_O_4_, and N-CNTs, is able to synergistically overcome the drawbacks of the individual components, such as high redox activity, porous structure with ion-accessible sites, and high electrical conductivity. The novel MIL-88A/NiCo_2_O_4_@N-CNT composite is shown to possess excellent electrochemical kinetics and shows great promise to be used as an excellent electrode material for future advanced energy storage applications.

**Figure 6:**
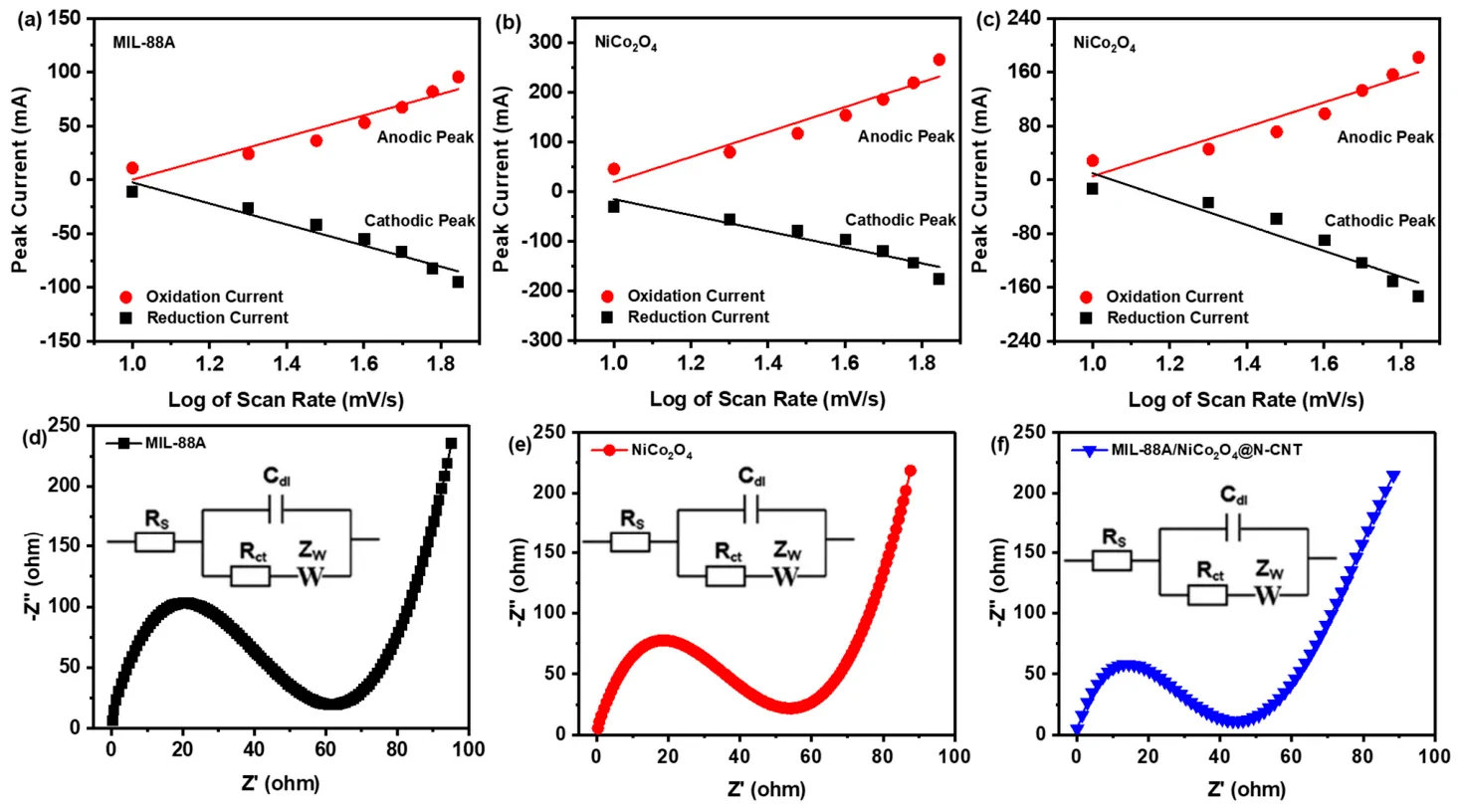
Electrochemical kinetic and impedance characteristics of the prepared electrodes: dependence of anodic and cathodic peak currents on the logarithm of scan rate for (**a**) MIL-88A, (**b**) NiCo_2_O_4_, and (**c**) MIL-88A/NiCo_2_O_4_@N-CNT; Nyquist plots and corresponding equivalent electrical circuits of (**d**) MIL-88A, (**e**) NiCo_2_O_4_, and (**f**) MIL-88A/NiCo_2_O_4_@N-CNT.

For the sake of understanding reaction kinetics and charge storage mechanisms of synthesized electrodes, the dependence of anodic/cathodic peak currents on the scan rate has been studied based on the CV data. The linear fits of MIL-88A, NiCo_2_O_4_, and MIL-88A/NiCo_2_O_4_@N-CNT electrodes are presented in Figure 6a–c, respectively. The anodic and cathodic peak currents exhibit a linear dependence on the square root of the scan rate, indicating that the electrochemical reactions are strongly influenced by ion diffusion processes.

The peak current dependence can be described by the Randles–Ševčík relationship:

(16) $$i_{p}=k_{1}v^{1/2}$$

where $i_{p}$ represents the anodic or cathodic peak current, v is the scan rate, and $k_{1}$ is a constant related to the diffusion coefficient and electroactive species concentration. A linear relationship between peak current and scan rate indicates surface-controlled behavior, whereas a linear relationship with the square root of scan rate suggests diffusion-controlled charge storage processes.

Anodic and cathodic peak currents of the MIL-88A electrode (Figure 6a) both increase with increasing scan rate, consistent with reversible faradaic reactions (on Fe-based active sites). However, the relatively lower slope of the fitting curves indicates slower electrochemical kinetics due to the limited electrical conductivity of the MOF framework. In contrast, the NiCo_2_O_4_ electrode (Figure 6b) shows much greater anodic and cathodic peak current responses than does MIL-88A. This improved behavior is enabled by the multiple reversible redox reactions of Ni and Co ions that offer many sites for faradaic charge storage. The linear correlation between peak current and scan rate indicates that NiCo_2_O_4_ is well involved in the electrochemical reaction process. As shown in Figure 6c, the peak current response of the MIL-88A/NiCo_2_O_4_@N-CNT electrode is the highest, and the linear fitting behavior is also the best among all of the electrodes. The synergism of the MIL-88A, NiCo_2_O_4_, and N-CNTs leads to the enhancement of the anodic and cathodic currents. The porous structure of MIL-88A makes it possible to obtain good diffusion of the electrolyte; NiCo_2_O_4_ possesses a large number of redox-active sites, while N-CNTs have high electronic conductivity and a fast charge transfer rate. Therefore, the hybrid architecture promotes faster electrochemical reactions and improved utilization of active material.

EIS technique was used to determine charge transfer properties and internal resistance of synthesized electrodes. The performance of the Nyquist plots and equivalent circuits is illustrated in Figure 6d–f. The equivalent circuit was described to match the impedance spectra and consisted of the solution resistance (R_s_), charge transfer resistance (Rct), double-layer capacitance (Cdl), and Warburg impedance (Zw). Rct is the diameter of the semicircle, and R_s_ is the high-frequency intercept. The MIL-88A electrode showed an R_s_ value of 2.8 Ω and an Rct value of 18.5 Ω. The higher Rct value indicates slower electron transfer kinetics due to the poor electrical conductivity of the MOF framework. The NiCo_2_O_4_ electrode exhibited lower resistance values, with R_s_ = 2.4 Ω and Rct = 11.2 Ω. This improvement is related to the mixed-valence nature of NiCo_2_O_4_. The Ni^2+^/Ni^3+^ and Co^2+^/Co^3+^ redox couples facilitate electron transport and improve charge transfer kinetics. The MIL-88A/NiCo_2_O_4_@N-CNT electrode displayed the lowest impedance response. It showed an R_s_ value of 1.6 Ω and an Rct value of 5.4 Ω. The fitted impedance parameters are summarized in Table 1. The reduced Rct confirms faster interfacial charge transfer. This behavior is attributed to the ability of the conductive N-CNT network to offer effective channels for electron transfer. Furthermore, the hybrid electrode has improved porosity, which promotes the diffusion of the electrolyte towards the active sites. The results show that the electrochemical reaction is better than that of each component in the MIL-88A/NiCo_2_O_4_@N-CNT architecture.

**Figure 7:**
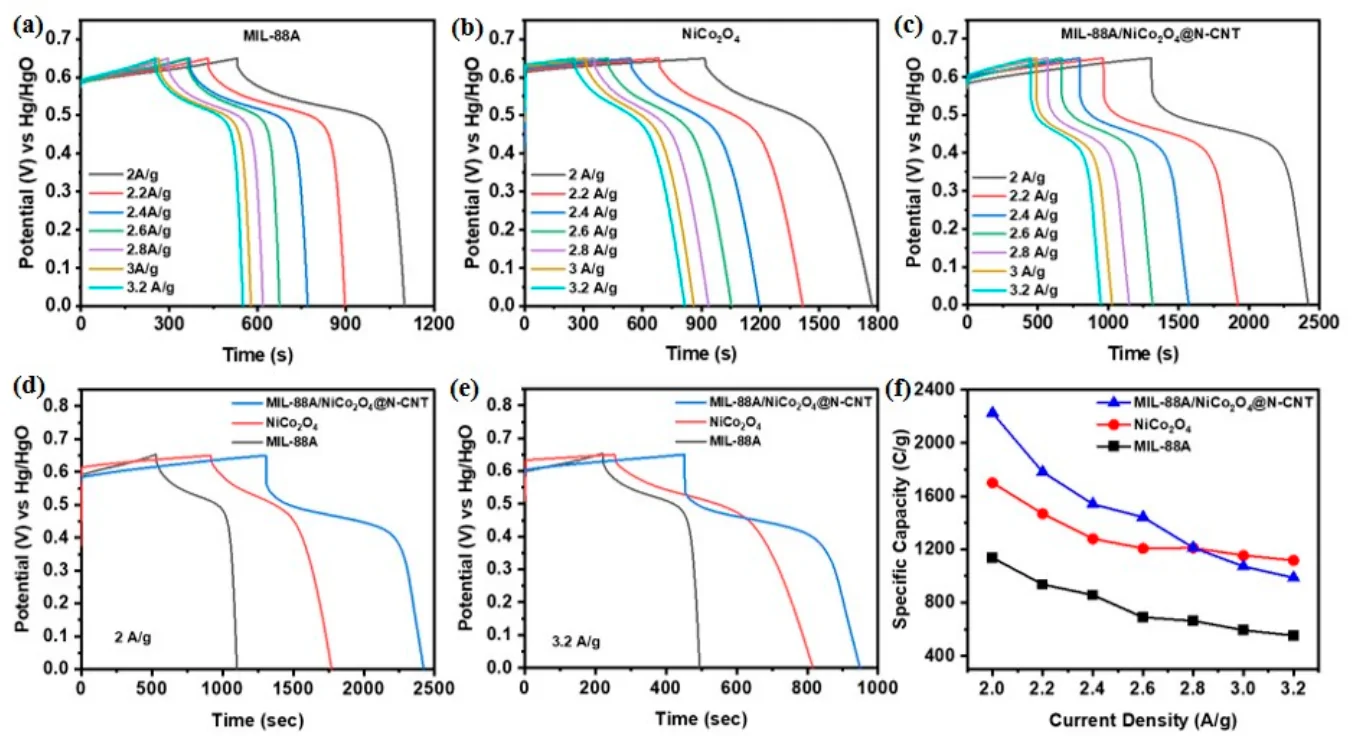
The profiles of different current density electrodes (2.0–3.2 A g^−1^) of the MIL-88A, NiCo_2_O_4_, and MIL-88A/NiCo_2_O_4_@N-CNT are shown as (**a**), (**b**), and (**c**), respectively; comparative GCD curves of the three electrodes at (**d**) 2.0 A g^−1^ and (**e**) 3.2 A g^−1^; and (**f**) specific capacity as a function of current density for MIL-88A, NiCo_2_O_4_, and MIL-88A/NiCo_2_O_4_@N-CNT electrodes.

The charge storage behavior of the prepared electrodes was further investigated by galvanostatic charge–discharge (GCD) measurements. The GCD profiles of MIL-88A, NiCo_2_O_4_, and MIL-88A/NiCo_2_O_4_@N-CNT electrodes at different current densities (2.0–3.2 A g^−1^) are shown in Figure 7a–c. All electrodes exhibit nonlinear charge–discharge profiles, indicating the contribution of faradaic redox reactions and confirming their pseudocapacitive behavior. The discharge time of all electrodes decreases with increasing current density due to limited ion diffusion at higher charge–discharge rates. Among the three electrodes, the MIL-88A/NiCo_2_O_4_@N-CNT electrode shows the longest discharge duration at all current densities, indicating higher charge storage capability. This improved performance is attributed to the synergistic effect of the porous MIL-88A framework, redox-active NiCo_2_O_4_ nanoparticles, and conductive N-CNT network.

The comparative GCD curves at 2.0 and 3.2 A g^−1^ are shown in Figure 7d,e. The hybrid electrode exhibits a longer discharge time compared with pristine MIL-88A and NiCo_2_O_4_ electrodes, confirming enhanced utilization of electroactive sites and improved electron/ion transport kinetics. As shown in Figure 7f, the specific capacity decreases gradually with increasing current density due to reduced ion diffusion time and incomplete utilization of active sites at higher current loads. The MIL-88A/NiCo_2_O_4_@N-CNT electrode exhibits the highest specific capacity over the entire current density range, followed by NiCo_2_O_4_ and MIL-88A electrodes. At the lowest current density of 2.0 A g^−1^, the specific capacities are approximately 1189 C g^−1^ for MIL-88A, 1703 C g^−1^ for NiCo_2_O_4_, and 2209 C g^−1^ for MIL-88A/NiCo_2_O_4_@N-CNT, respectively. Superior capacity of the composite electrode is achieved due to the synergistic effect of all three components of the hybrid electrode. The porous nature of the MIL-88A structure allows the penetration of the electrolyte, the NiCo_2_O_4_ structure provides numerous active Ni and Co redox sites, and the N-CNT structure provides high electrical conductivity and facilitates electron transfer. The specific capacities of the prepared electrodes, determined by CV and GCD data, are given in Table 2.

### 4.2. Asymmetric Supercapacitor Device Performance

**Figure 8:**
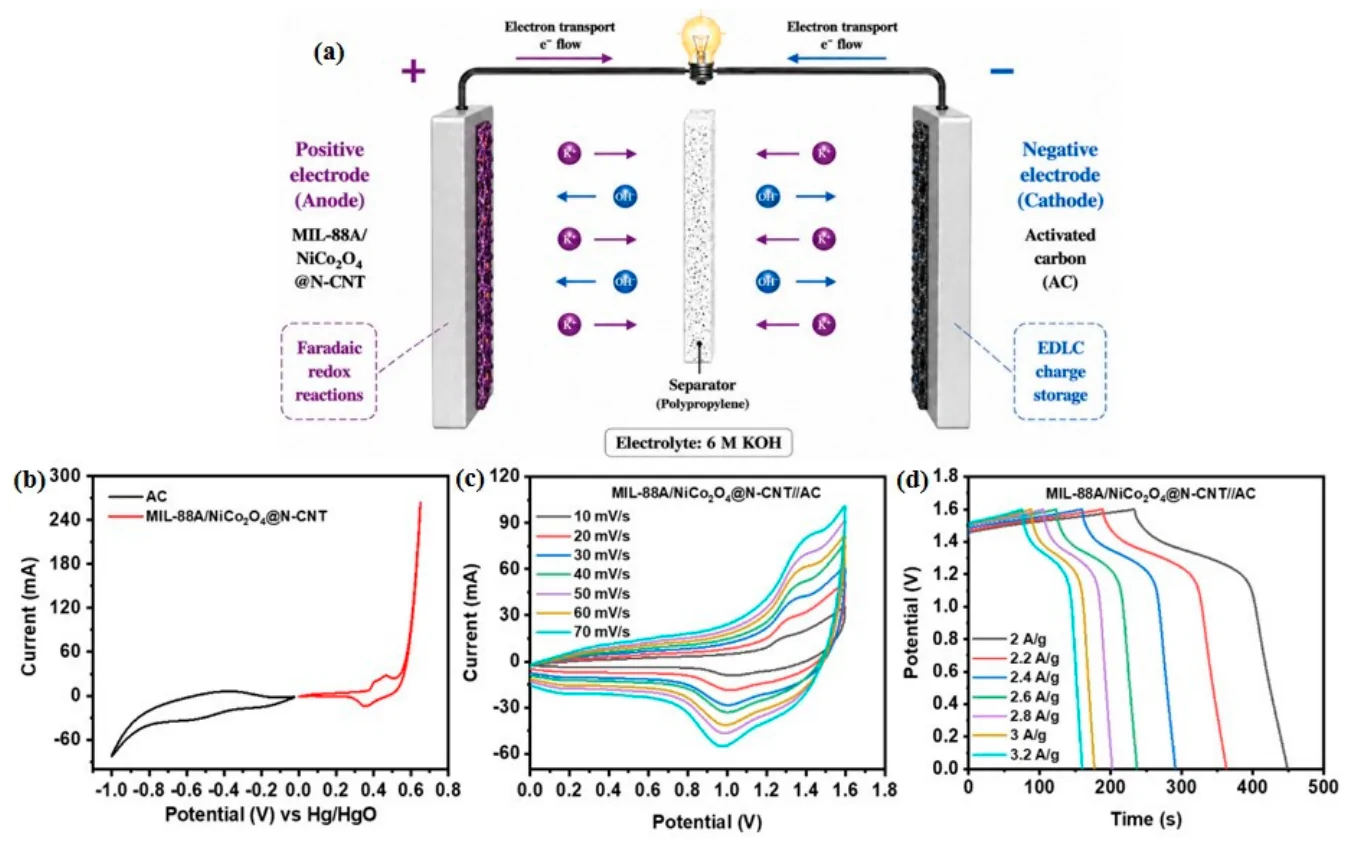
(**a**) Schematic diagram of the MIL-88A/NiCo_2_O_4_@N-CNT//AC hybrid composite energy storage device, (**b**) CV analysis of MIL-88A/NiCo_2_O_4_@N-CNT//AC and AC at 10 mV s^−1^, and (**c**,**d**) CV and GCD analyses of the assembled MIL-88A/NiCo_2_O_4_@N-CNT//AC device at different scan rates and current densities, respectively.

To test the feasibility of the fabricated hybrid electrode for energy storage, an asymmetric supercapacitor was fabricated, consisting of MIL-88A/NiCo_2_O_4_@N-CNT and activated carbon (AC) as the positive and negative electrodes, respectively, in KOH electrolyte. The design and schematics of the assembled asymmetric device and ion storage are shown in Figure 8a. The charge balance between the positive and negative electrodes was kept as follows:

The electrochemical behavior of individual electrodes was first compared by CV measurements at 10 mV s^−1^ (Figure 8b). The MIL-88A/NiCo_2_O_4_@N-CNT electrode exhibits a pronounced redox response, confirming its faradaic charge storage mechanism, while the CV profile of the AC electrode demonstrates an almost rectangular shape indicating the presence of EDLC. The combination of the battery-type positive electrode and capacitive negative electrode enables an extended operating voltage window and improved energy storage performance.

The CV of the constructed MIL-88A/NiCo_2_O_4_@N-CNT//AC recorded at different scan rates (10–70 mV s^−1^) is shown in Figure 8c. The electrochemical reversibility and fast charge transfer rates are observed with an increase in scan rate. Although peak currents increase with the growth of scan rate, the general shape of the CV curve remains unchanged, which proves efficient ion diffusion and good rate capabilities of the assembled device. GCD curves of the asymmetric device recorded at current densities from 2.0 to 3.2 A g^−1^ are given in Figure 8d. The presence of a reversible Faradaic reaction at the positive electrode is shown by non-linear discharge curves. Nearly symmetric shapes of charge–discharge curves indicate good reversibility and low polarization. With increasing current density, the discharge time gradually decreases due to limited ion diffusion and reduced active-site utilization at higher rates. The non-linear GCD profiles are consistent with the battery-type/Faradaic contribution of the positive electrode. Therefore, the reported energy density represents a capacity-based estimate derived from the experimentally measured discharge charge and the operating voltage window, rather than an energy value obtained from an ideal linear-capacitor approximation. The overall electrochemical response confirms that the MIL-88A/NiCo_2_O_4_@N-CNT//AC asymmetric device benefits from the synergistic combination of the porous MIL-88A framework, redox-active NiCo_2_O_4_ component, conductive N-CNT network, and AC negative electrode. These structural advantages promote rapid electron transport, efficient ion migration, and enhanced energy storage performance.

**Figure 9:**
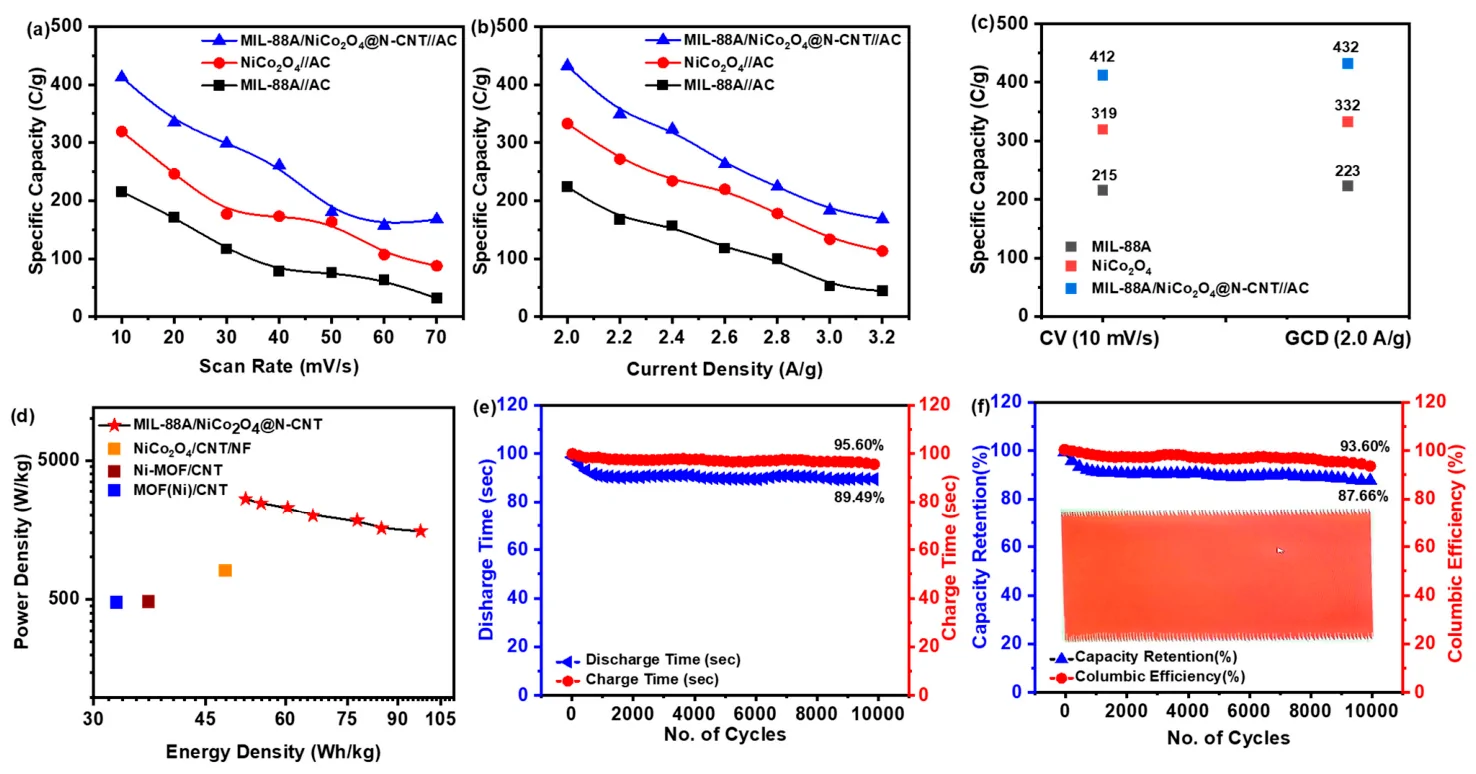
Electrochemical performance of the asymmetric energy storage devices: (**a**) specific capacity as a function of scan rate (10–70 mV s^−1^), (**b**) specific capacity as a function of current density (2.0–3.2 A g^−1^), (**c**) comparison of specific capacities obtained from CV at 10 mV s^−1^ and GCD at 2.0 A g^−1^ for MIL-88A//AC, NiCo_2_O_4_//AC, and MIL-88A/NiCo_2_O_4_@N-CNT//AC, (**d**) Ragone plot showing the ED and power density of the MIL-88A/NiCo_2_O_4_@N-CNT//AC device, (**e**) charge- and discharge-time retention over 10,000 cycles, and (**f**) capacity retention and coulombic efficiency of the MIL-88A/NiCo_2_O_4_@N-CNT//AC device over 10,000 cycles.

The practical energy storage performance of the prepared electrodes was further evaluated by assembling asymmetric devices using MIL-88A, NiCo_2_O_4_, and MIL-88A/NiCo_2_O_4_@N-CNT as positive electrodes coupled with activated carbon (AC) as the negative electrode. The electrochemical performance of the assembled devices is presented in Figure 9. The specific capacities obtained from CV measurements at 10 mV s^−1^ are compared in Figure 9a,c. The MIL-88A//AC, NiCo_2_O_4_//AC, and MIL-88A/NiCo_2_O_4_@N-CNT//AC devices deliver specific capacities of approximately 215, 319, and 412 C g^−1^, respectively. The hybrid device exhibits the highest capacity, demonstrating the advantage of integrating MIL-88A, NiCo_2_O_4_, and N-CNT into a single electrode architecture. The enhanced capacity originates from the combined contribution of the porous MIL-88A framework, abundant redox-active sites of NiCo_2_O_4_, and improved electrical conductivity provided by the N-CNT network. The rate capability of the asymmetric devices was further investigated through GCD measurements at different current densities (2.0–3.2 A g^−1^) as shown in Figure 9b,c. The specific capacities obtained at 2.0 A g^−1^ are 223 C g^−1^ for MIL-88A//AC, 332 C g^−1^ for NiCo_2_O_4_//AC, and 432 C g^−1^ for MIL-88A/NiCo_2_O_4_@N-CNT//AC, respectively. The higher capacity of the hybrid device confirms improved utilization of electroactive materials and faster charge transfer kinetics.

The Ragone plot (Figure 9d) was used to estimate the energy storage performance of the MIL-88A/NiCo_2_O_4_@N-CNT//AC device. The maximum ED of 98.6 Wh kg^−1^ at a power density of 1727 W kg^−1^ is achieved by the device. The synergistic effect between the Faradaic positive electrode and capacitive AC negative electrode is responsible for good energy storage performance of the asymmetric device. The hierarchical porous structure facilitates ion diffusion, while the conductive N-CNT network improves electron transport during charge–discharge processes. The electrochemical activity of single electrodes was first characterized by CVs at 10 mV s^−1^ (Figure 8b). The cycling stability of the assembled device was investigated for 10,000 charge–discharge cycles. The device exhibits superb structural and electrochemical stability in repeated cycling, as demonstrated in Figure 9e,f. The device maintains 87.66% capacity after 10,000 cycles and has a Coulombic efficiency of 93.60%. The stable cycling performance suggests a good reversibility of the redox reactions and effective structural stabilization of the hybrid electrode design. It should be noted that capacity retention and coulombic efficiency describe different aspects of cycling stability. The capacity retention reflects preservation of the accessible charge storage capability relative to the initial state, whereas the coulombic efficiency represents the reversibility of charge transfer within an individual charge–discharge cycle. The coulombic efficiency of 93.60% after prolonged cycling suggests modest electrochemical irreversibility, which may arise from polarization, internal resistance, incomplete reversibility of Faradaic reactions, ion trapping within the porous electrode structure, and minor parasitic interfacial processes. Nevertheless, the retention of 87.66% of the initial capacity after 10,000 cycles demonstrates that most electrochemically active sites remain accessible and that the hybrid electrode architecture retains substantial long-term stability. The overall performance of the MIL-88A/NiCo_2_O_4_@N-CNT//AC asymmetric device shows high capacity, high ED, and high cycling stability, suggesting that it is a promising, efficient electrode system for other energy storage applications.

The comparative specific capacities of the assembled asymmetric devices obtained from CV and GCD measurements are summarized in Table 3, while the key device-level energy storage and cycling parameters of the MIL-88A/NiCo_2_O_4_@N-CNT//AC device are presented in Table 4. The comparison in Table 5 demonstrates that the MIL-88A/NiCo_2_O_4_@N-CNT//AC device achieves a competitive energy storage performance compared with previously reported MOF-, NiCo_2_O_4_-, and CNT-based asymmetric supercapacitors.

### 4.3. Charge Storage Kinetics Analysis of MIL-88A/NiCo_2_O_4_@N-CNT//AC Asymmetric Device

**Figure 10:**
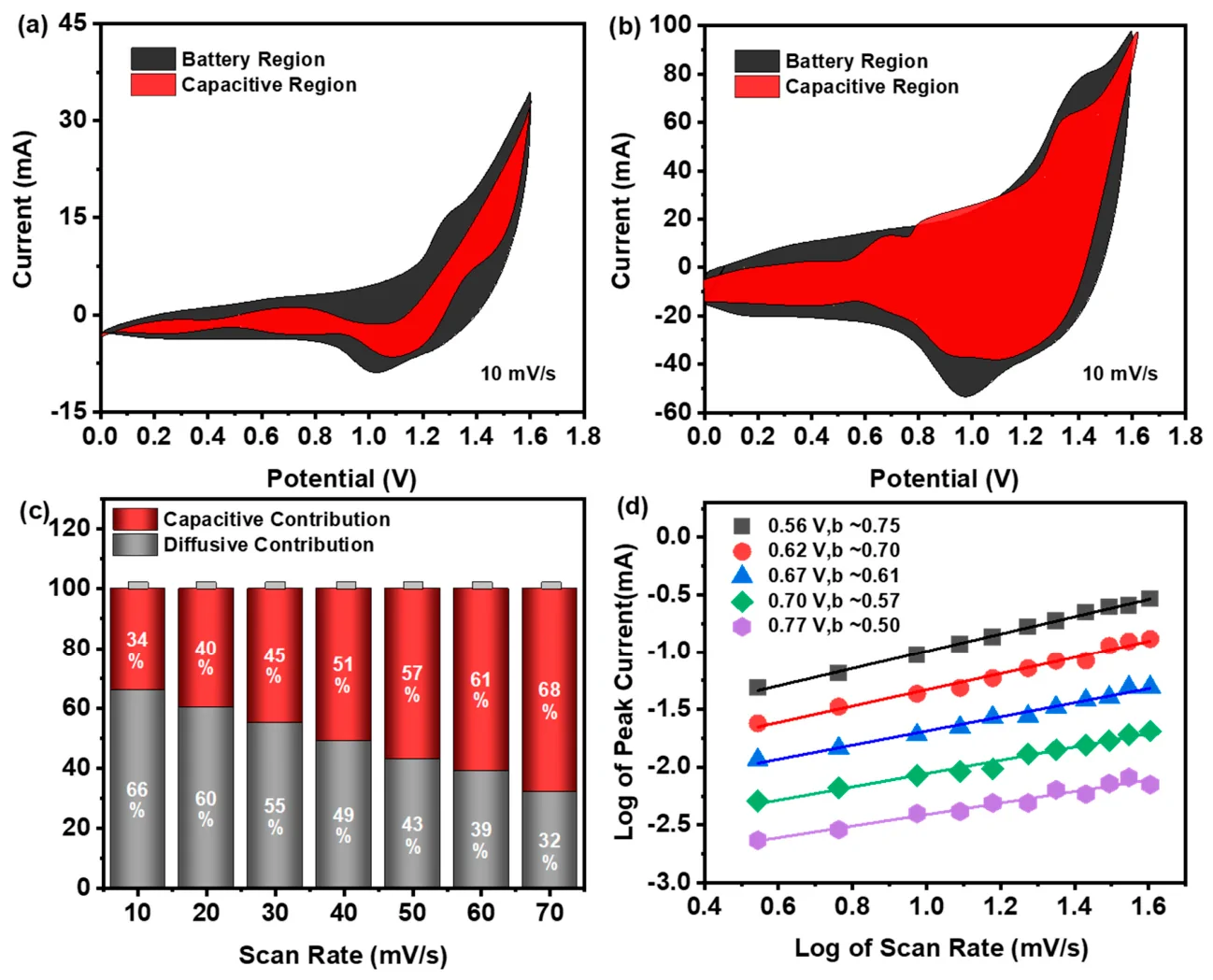
Charge storage kinetics of the MIL-88A/NiCo_2_O_4_@N-CNT//AC asymmetric device based on Dunn’s model: (**a**,**b**) capacitive and diffusion-controlled contributions to the CV response at different scan rates, (**c**) percentage contributions of capacitive and diffusion-controlled charge storage at scan rates from 10 to 70 mV s^−1^, and (**d**) corresponding kinetic analysis illustrating the scan-rate-dependent charge storage behavior of the hybrid device.

The charge storage mechanism of the MIL-88A/NiCo_2_O_4_@N-CNT//AC asymmetric device was further investigated using the Dunn’s model. The separated capacitive and diffusion-controlled contributions at different scan rates are presented in Figure 10a,b. The CV responses were analyzed at scan rates ranging from 10 to 70 mV s^−1^. The results reveal the coexistence of both surface-controlled and diffusion-controlled charge storage processes in the hybrid device. At higher scan rates, capacitive storage increases gradually, showing a higher rate of the surface reactions and better accessibility of the electroactive sites at high-rate operation. At various scan rates, the quantitative contribution of capacitive and diffusion-controlled processes was computed, as illustrated in Figure 10c. In the case of 10 mV s^−1^, the capacitive contribution is 34%, and the diffusion-controlled contribution dominates. With increasing scan rate, the capacitive contribution continuously increases, reaching 40%, 45%, 51%, 57%, 61%, and 68% at 20, 30, 40, 50, 60, and 70 mV s^−1^, respectively. Likewise, at the same scan rates, the diffusion-controlled contribution drops to 60%, 55%, 49%, 43%, 39%, and 32%. The gradual transition suggests that ion diffusion is prominent at low scan rates because the electrolyte can penetrate the porosities of the material, and the surface-controlled process is more significant at high scan rates because the electron transfer reaction and ion adsorption/desorption are fast.

The b value analysis was further used to assess the scan rate-dependent kinetic behavior (Figure 10d). The data of peak current and scan rate were analyzed by the log (i)–log (v) fitting method. The calculated b-values of the anodic and cathodic peaks are about 0.82 and 0.76, respectively, showing a mixed charge storage mechanism, reflecting a high capacitive component. The b-values between 0.5 and 1.0, which are indicative of diffusion-controlled faradaic reactions and surface-controlled capacitive storage, strongly suggest that both processes are occurring simultaneously. The relatively high b-values are indicative of better reaction kinetics and a fast charge transfer process in the hybrid electrode. The synergistic structure of the MIL-88A/NiCo_2_O_4_@N-CNT hybrid electrode is responsible for the improved kinetic response. The multiple oxidation states of NiCo_2_O_4_ introduce a large number of redox-active sites into the structure, and the porous MIL-88A structure enables the diffusion of electrolyte ions through the structure. The interconnected N-CNT network enhances the electrical conductivity and creates continuous electron transfer pathways. Therefore, the hybrid structure effectively balances diffusion-controlled and capacitive charge storage mechanisms, resulting in improved rate capability and electrochemical performance of the asymmetric device.

### 4.4. HER Electrocatalytic Performance of MIL-88A/NiCo_2_O_4_@N-CNT Hybrid Catalyst

**Figure 11:**
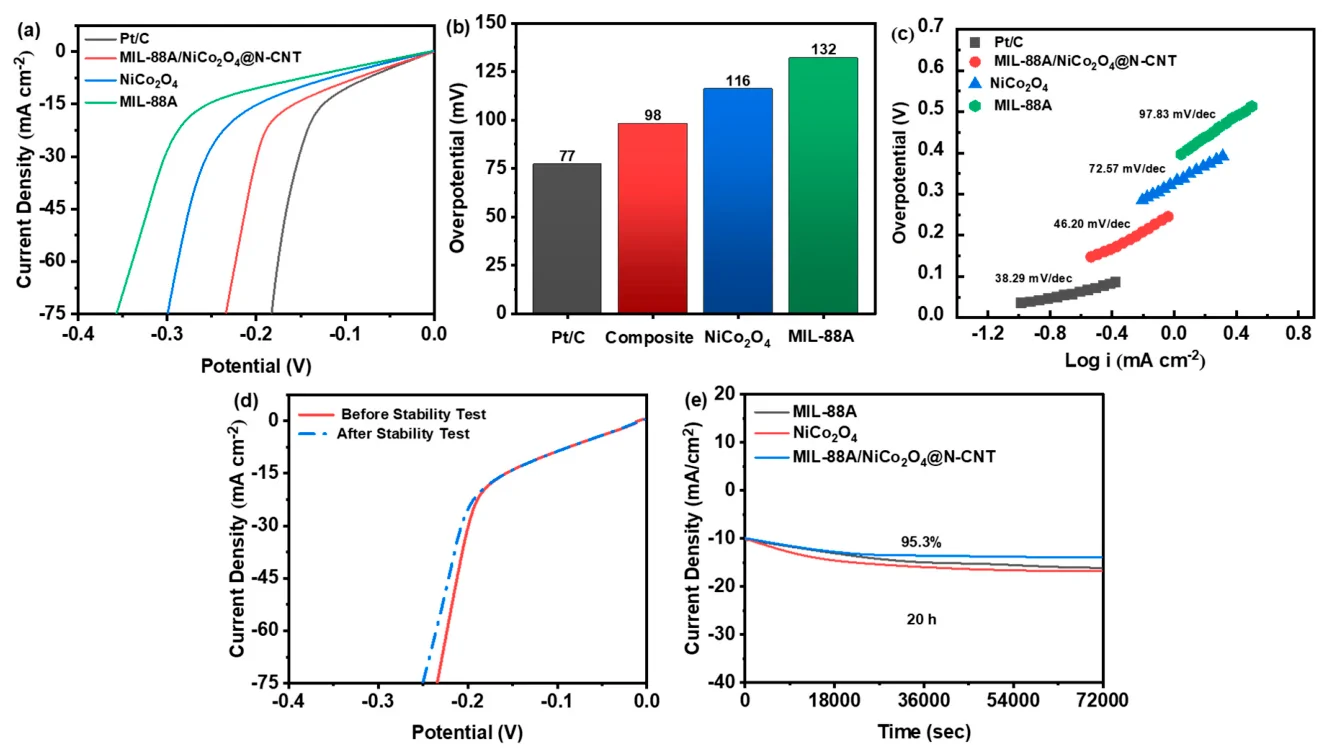
HER electrocatalytic performance of the prepared catalysts: (**a**) LSV polarization curves of Pt/C, MIL-88A, NiCo_2_O_4_, and MIL-88A/NiCo_2_O_4_@N-CNT, (**b**) comparison of overpotential values, (**c**) corresponding Tafel plots and Tafel slopes, (**d**) LSV curves of MIL-88A/NiCo_2_O_4_@N-CNT before and after the stability test, and (**e**) long-term electrocatalytic stability test.

The synthesized catalysts were tested for their HER activity using LSV, Tafel analysis, and stability tests. Recent reviews of Ni-based nanohybrid electrocatalysts have shown that coupling Ni-containing active phases with conductive supports can improve interfacial charge transfer, electron/ion transport, surface accessibility, and the utilization of catalytic sites during electrocatalytic water splitting [44]. The catalytic performance of MIL-88A, NiCo_2_O_4_, MIL-88A/NiCo_2_O_4_@N-CNT, and commercial Pt/C catalysts in the alkaline electrolyte is shown in Figure 11. The LSV polarization curves (Figure 11a) reveal significant differences in HER activity among the investigated catalysts. The MIL-88A/NiCo_2_O_4_@N-CNT hybrid catalyst exhibits much improved HER performance compared with pristine MIL-88A and NiCo_2_O_4_ electrodes. The polarization curve of the hybrid catalyst is shifted toward lower overpotential, indicating faster hydrogen evolution kinetics. As expected, Pt/C shows the highest activity; however, the MIL-88A/NiCo_2_O_4_@N-CNT catalyst demonstrates competitive HER performance among the synthesized materials. The overpotentials needed to obtain a current density of 10 mA cm^−2^ are tested and compared in Figure 11b. The overpotentials for the following are 77 mV for Pt/C, 98 mV for MIL-88A/NiCo_2_O_4_@N-CNT, 116 mV for NiCo_2_O_4_, and 132 mV for MIL-88A. The construction of the MIL-88A/NiCo_2_O_4_@N-CNT hybrid composite is found to be effective in enhancing the HER activity. This synergistic effect between the three components of the catalyst can be the reason for its enhanced catalytic performance. The porous MIL-88A framework offers plenty of channels for the electrolyte to penetrate and open up the active sites. There are several catalytic centers linked to the Ni/Co redox states in NiCo_2_O_4_. In the meantime, the N-CNT network enhances the electrical conductivity and promotes electron transport during the HER process.

The Tafel plots (Figure 11c) were also employed to analyze the HER reaction kinetics. The calculated Tafel slopes are 38.29 mV dec^−1^ for Pt/C, 46.20 mV dec^−1^ for MIL-88A/NiCo_2_O_4_@N-CNT, 72.57 mV dec^−1^ for NiCo_2_O_4_, and 97.83 mV dec^−1^ for MIL-88A. The Tafel slope of the MIL-88A/NiCo_2_O_4_@N-CNT catalyst is significantly smaller than that of the individual components, suggesting higher reaction rate constants for HER and more favorable reaction pathways. The lower slope indicates that there are faster electron transfer reactions and better coupling of the catalyst surface with the electrolyte ions. The stability of MIL-88A/NiCo_2_O_4_@N-CNT was then tested using the LSV polarization curves before and after stability testing (Figure 11d). Under continuous operation, the polarization curve exhibits only a slight change, indicating the excellent structural stability of the hybrid catalyst. In addition, the chronoamperometric plot (Figure 11e) shows stable performance of the HER operation for 20 h at a constant current density of ~10 mA cm^−2^. After the durability test, the catalyst retains 95.3% of its current, thereby demonstrating excellent catalytic stability.

In conclusion, the rationally designed hybrid architecture of MIL-88A/NiCo_2_O_4_@N-CNT is responsible for its superior HER performance. This porous MIL-88A structure allows for easy access of the electrolyte, NiCo_2_O_4_ offers rich active catalytic sites, and N-CNT creates conductive pathways for rapid electron transport. These components are strongly coupled to increase the catalytic efficiency and retain structural stability during extended electrochemical operation. Overall, the MIL-88A/NiCo_2_O_4_@N-CNT hybrid catalyst exhibits a low overpotential of 98 mV at 10 mA cm^−2^, a small Tafel slope of 46.20 mV dec^−1^, and excellent durability with 95.3% current retention after 20 h, demonstrating its potential as an efficient bifunctional electrode material for energy conversion applications. The HER performance comparison presented in Table 6 and Table 7 demonstrates that the MIL-88A/NiCo_2_O_4_@N-CNT catalyst exhibits superior catalytic activity compared with previously reported NiCo_2_O_4_-, MOF-, and carbon-supported catalysts.

## 5. Conclusions

In this study, a hierarchical MIL-88A/NiCo_2_O_4_@N-CNT hybrid composite was successfully designed and synthesized by integrating a porous Fe-based MOF framework, redox-active NiCo_2_O_4_, and a conductive N-CNT network. The structural, morphological, and surface analyses were in good agreement with the successful synthesis of the hybrid architecture, which enhanced not only the surface accessibility and electron transport but also the structural stability, due to the synergistic interaction of the three components. The composite had an improved BET surface area of 176.43 m^2^ g^−1^, thus making it more porous for the penetration of electrolyte and providing more electroactive sites. Electrochemical investigations showed that the MIL-88A/NiCo_2_O_4_@N-CNT electrode exhibited enhanced charge storage performance compared with that of the pristine NiCo_2_O_4_ and MIL-88A electrodes, with specific capacities of 2036 C g^−1^ at 10 mV s^−1^ and 2209 C g^−1^ at 2.0 A g^−1^, respectively. The assembled MIL-88A/NiCo_2_O_4_@N-CNT//AC asymmetric supercapacitor exhibited excellent energy storage capability, providing specific capacities of 412 C g^−1^ (CV) and 432 C g^−1^ (GCD), with a high ED of 98.6 Wh kg^−1^ at a power density of 1727 W kg^−1^. Furthermore, the supercapacitor showed excellent long-term stability with 87.66% of the energy being preserved after 10,000 cycles and a coulombic efficiency of more than 93.6%. The hybrid composite also showed promising HER electrocatalytic activity, requiring only 98 mV overpotential at 10 mA cm^−2^, with a low Tafel slope of 46.20 mV dec^−1^ and 95.3% current retention after 20 h of continuous operation. Kinetic analysis confirmed the coexistence of capacitive and diffusion-controlled charge storage processes, highlighting the favorable charge transfer behavior of the hybrid electrode. The enhanced performance is attributed to the complementary roles of MIL-88A in providing porous ion-accessible channels, NiCo_2_O_4_ in supplying abundant redox-active centers, and N-CNTs in establishing efficient conductive pathways. Overall, this work demonstrates that the rational integration of MOF, transition-metal oxide, and carbon nanostructures provides an effective strategy for developing multifunctional electrode materials for advanced asymmetric supercapacitors and hydrogen evolution applications.

## Figures and Tables

**Table 1:** Electrochemical impedance parameters (Rs and Rct) of MIL-88A, NiCo_2_O_4_, and MIL-88A/NiCo_2_O_4_@N-CNT electrodes.

Sample	Rs (Ω)	Rct (Ω)
MIL-88A	2.8	18.5
NiCo_2_O_4_	2.4	11.2
MIL-88A/NiCo_2_O_4_@N-CNT	1.6	5.4

**Table 2:** Comparison of specific capacity obtained from CV and GCD measurements.

Electrode	Specific Capacity 10 mV s^−1^ (C g^−1^)	Specific Capacity at 2.0 A g^−1^ (C g^−1^)
MIL-88A	1113	1189
NiCo_2_O_4_	1596	1703
MIL-88A/NiCo_2_O_4_@N-CNT	2036	2209

**Table 3:** Comparison of electrochemical performance of MIL-88A//AC, NiCo_2_O_4_//AC, and MIL-88A/NiCo_2_O_4_@N-CNT//AC asymmetric devices.

Asymmetric Device	Specific Capacity from CV at 10 mV s^−1^ (C g^−1^)	Specific Capacity from GCD at 2.0 A g^−1^ (C g^−1^)
MIL-88A//AC	215	223
NiCo_2_O_4_//AC	319	332
MIL-88A/NiCo_2_O_4_@N-CNT//AC	412	432

**Table 4:** Device-level energy storage and cycling performance of MIL-88A/NiCo_2_O_4_@N-CNT//AC.

Device	Energy Density (Wh kg^−1^)	Power Density (W kg^−1^)	Capacity Retention After 10,000 Cycles (%)	Coulombic Efficiency After 10,000 Cycles (%)
MIL-88A/NiCo_2_O_4_@N-CNT//AC	98.6	1727	87.66	93.6

**Table 5:** Comparison of MIL-88A/NiCo_2_O_4_@N-CNT//AC asymmetric supercapacitor with reported MOF-, NiCo_2_O_4_-, and CNT-based hybrid devices.

Electrode Material (Positive Electrode)	Energy Density (Wh kg^−1^)	Power Density (W kg^−1^)	Cycling Stability	Ref.
MIL-88A/CNT hybrid electrode	~100	—	80.1% after 15,000 cycles	[15]
NiCo_2_O_4_/CNT/NF core–shell network	48.3	799.9	78.9% after 2000 cycles	[34]
Porous NiCo_2_O_4_ microspheres	19.1	1839	—	[35]
Ni-MOF/CNT composite	36.6	480	95% after 5000 cycles	[36]
Ni-MOF@CNT/graphene-Ni foam	0.3396 mWh cm^−2^	—	93% after 4000 cycles	[37]
NiMOF/CNT nanosheets	113.8	800	87.7% rate retention	[38]
NiO/Ni/NCNT derived from Ni-MOF	33.89	800	~90% after 40,000 cycles	[39]
NiO/C@CNF derived from Ni-MOF	58.43	1947	~88% after 5000 cycles	[40]
MOF(Ni)/CNT layered composite	32.6	476.5	83.2% after 5000 cycles	[41]
CNT@NiO/MOF-derived porous carbon	25.4	400	93% after 10,000 cycles	[42]
CNT/C/NiMoO_4_ composite	32.6	150.5	96.5% after 1500 cycles	[43]
MIL-88A/NiCo_2_O_4_@N-CNT	98.6	1727	87.66% after 10,000 cycles	This work

**Table 6:** HER electrocatalytic performance comparison of prepared catalysts.

Catalyst	Overpotential at 10 mA cm^−2^ (mV)	Tafel Slope (mV dec^−1^)
Pt/C	77	38.29
MIL-88A	132	97.83
NiCo_2_O_4_	116	72.57
MIL-88A/NiCo_2_O_4_@N-CNT	98	46.2

**Table 7:** Comparison of HER electrocatalytic performance of MIL-88A/NiCo_2_O_4_@N-CNT with previously reported MOF-, NiCo_2_O_4_-, and carbon-based catalysts.

Electrocatalyst	Electrolyte	Overpotential at 10 mA cm^−2^ (mV)	Tafel Slope (mV dec^−1^)	Stability	Ref.
NiCo_2_O_4_ nanosheets	1 M KOH	260	98	12 h	[45]
NiCo_2_O_4_ nanoparticles	1 M KOH	210	82	10 h	[46]
NiCo_2_O_4_/N-doped carbon composite	1 M KOH	180	75	20 h	[47]
CNT-supported NiCo_2_O_4_	1 M KOH	165	68	10 h	[48]
NiCo_2_O_4_@N-CNT hybrid	1 M KOH	145	61	20 h	[49]
NiCo_2_O_4_/rGO composite	1 M KOH	150	70	12 h	[50]
Ni-MOF derived Ni/N-C catalyst	1 M KOH	160	72	24 h	[51]
Co-MOF derived N-doped carbon catalyst	1 M KOH	170	79	10 h	[52]
MOF-derived CoNi alloy/N-C catalyst	1 M KOH	120	55	24 h	[53]
NiCo_2_O_4_@carbon nanotube composite	1 M KOH	135	58	20 h	[54]
NiCo_2_O_4_/g-C_3_N_4_ hybrid catalyst	1 M KOH	125	52	20 h	[50]
MOF-derived NiCo_2_O_4_/N-doped carbon	1 M KOH	110	49	20 h	[55]
Fe-based MOF-derived catalyst	1 M KOH	220	90	10 h	[56]
MIL-88A-based hybrid catalyst	1 M KOH	150	65	20 h	[57]
MIL-88A/NiCo_2_O_4_@N-CNT (this work)	1 M KOH	98	46.2	95.3% retention after 20 h	This work

## Data Availability

The data supporting the findings of this study are available upon reasonable request.

## Supplementary Materials

The following supporting information can be downloaded at: https://www.mdpi.com/article/10.3390/molecules31213829/s1. Figure S1: BJH pore-size distribution curve of MIL-88A/NiCo_2_O_4_@N-CNT derived from the N_2_ adsorption–desorption measurements.
