# Supplementary material for: Hierarchical MIL-88A/NiCo2O4@N-CNT Hybrid for Asymmetric Supercapacitors and Hydrogen Evolution Reaction
Source: Molecules. 2026 Oct 23;31(21):3829. doi: 10.3390/molecules31213829 (PMC13764863; doi:10.3390/molecules31213829)
Supplement: Supplementary file 1 [file molecules-31-03829-s001.zip › molecules-4597328-supplementary.pdf]

# Hierarchical MIL-88A/$NiCo_2O_4$@N-CNT Hybrid for Asymmetric Supercapacitors and Hydrogen Evolution Reaction

Jaffer Saddique[1,*], Muhammad Ahsan[1], Zhao Huihui[1], Marcin Gackowski[2], Łukasz Szeleszczuk[3,**], Amjad A. Almunyif[4]

1 Department of physics, Qilu institute of Technology, Jinan 250200, Shandong, China;
2 Department of Toxicology and Bromatology, L. Rydygier Collegium Medicum in Bydgoszcz, Nicolaus Copernicus University in Torun, 2 Jurasza Str., 85-089 Bydgoszcz, Poland;
3 Department of Organic and Physical Chemistry, Medical University of Warsaw, 1 Banacha Str., 02-097 Warsaw, Poland;
4 Department of Physics, College of Sciences, Princess Nourah bint Abdulrahman University, P.O. Box 84428, Riyadh 11671, Saudi Arabia.

* Correspondence: jafferphy@qlit.edu.cn; lukasz.szeleszczuk@wum.edu.pl

## Supporting Data

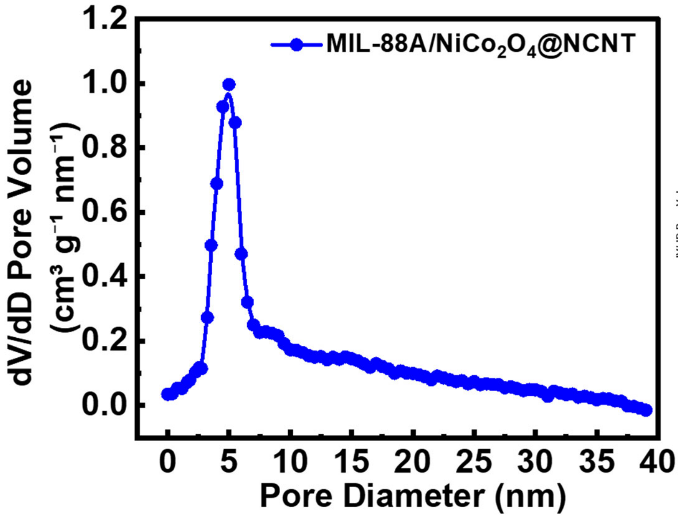


**Figure S1.** BJH pore-size distribution curve of MIL-88A/$NiCo_2O_4$@N-CNT derived from the $N_2$ adsorption–desorption measurements.
